# Differential Sensitivity of the Protein Translation Initiation Machinery and mTOR Signaling to *MECP2* Gain- and Loss-of-Function Involves MeCP2 Isoform-Specific Homeostasis in the Brain

**DOI:** 10.3390/cells11091442

**Published:** 2022-04-24

**Authors:** Marjorie Buist, Nada El Tobgy, Danilo Shevkoplyas, Matthew Genung, Annan Ali Sher, Shervin Pejhan, Mojgan Rastegar

**Affiliations:** Department of Biochemistry and Medical Genetics, Max Rady College of Medicine, Rady Faculty of Health Sciences, University of Manitoba, Winnipeg, MB R3E 0J9, Canada; buistm@myumanitoba.ca (M.B.); eltobgyn@myumanitoba.ca (N.E.T.); shevkopd@myumanitoba.ca (D.S.); genungm@myumanitoba.ca (M.G.); annan.sher@umanitoba.ca (A.A.S.); shervin.pejhan@lhsc.on.ca (S.P.)

**Keywords:** epigenetics, MeCP2 isoforms, MeCP2E1, gene transcription, BDNF, brain development, protein translation, RTT, mTOR signaling, *miR-132*

## Abstract

Eukaryotic gene expression is controlled at multiple levels, including gene transcription and protein translation initiation. One molecule with key roles in both regulatory mechanisms is methyl CpG binding protein 2 (MeCP2). *MECP2* gain- and loss-of-function mutations lead to Rett Syndrome and *MECP2* Duplication Syndrome, respectively. To study *MECP2* gain-of-function, we generated stably transduced human brain cells using lentiviral vectors for both *MECP2E1* and *MECP2E2* isoforms. Stable overexpression was confirmed by Western blot and immunofluorescence. We assessed the impact of MeCP2E1-E2 gain-of-function on the MeCP2 homeostasis regulatory network (*MECP2E1/E2-BDNF*/BDNF-*miR-132*), mTOR-AKT signaling, ribosome biogenesis, markers of chromatin structure, and protein translation initiation. We observed that combined co-transduction of MeCP2 isoforms led to protein degradation of MeCP2E1. Proteosome inhibition by MG132 treatment recovered MeCP2E1 protein within an hour, suggesting its induced degradation through the proteosome pathway. No significant change was detected for translation initiation factors as a result of MeCP2E1, MeCP2E2, or combined overexpression of both isoforms. In contrast, analysis of human Rett Syndrome brains tissues compared with controls indicated impaired protein translation initiation, suggesting that such mechanisms may have differential sensitivity to *MECP2* gain- and loss-of-function. Collectively, our results provide further insight towards the dose-dependent functional role of MeCP2 isoforms in the human brain.

## 1. Introduction

Gene regulation is a complex cellular process that is tightly controlled at multiple different layers [1]. These include the interaction of *cis*-regulatory elements with the binding of *trans*-acting factors, along with an array of epigenetic mechanisms including DNA methylation, energy-dependent chromatin remodeling, nucleosome positioning, modification of DNA and RNA molecules, and regulatory RNAs [2,3,4,5]. Production of functional proteins is further controlled at the level of protein translation. Recent research has highlighted the impact of genetic mutations in certain epigenetic factors influencing protein translation initiation and their upstream regulatory pathways, such as the mammalian target of rapamycin (mTOR). One such example of an epigenetic factor with a suggested role in protein translation initiation is methyl CpG-binding protein 2 (MeCP2) [6]. MeCP2 is an epigenetic factor that is encoded by the X-linked *MECP2* (OMIM 300005) gene, discovered 30 years ago [7]. Mouse models with *Mecp2* loss- and gain-of function-mutations display a broad range of gene expression changes in the brain [8,9,10]. In humans, de novo *MECP2* loss-of-function mutations cause Rett Syndrome (RTT) (MIM 312750) [11]. MeCP2 is a dose-dependent protein, and *MECP2* gene duplication or triplication causes *MECP2* Duplication Syndrome (MDS). While RTT patients are primarily females, MDS is commonly a male disorder observed in boys who are born from asymptomatic mothers carrying the same *MECP2* gain-of-function mutation [12,13]. There are two functional MeCP2 protein variants known as MeCP2E1 (MeCP2*α* or MeC2B) and MeCP2E2 (MeCP2*β* or MeCP2A) isoforms, produced by alternative splicing of exons 1 and 2 of the *Mecp2*/*MECP2* gene. MeCP2E2 was the original isoform discovered in 1992 [7]. The second isoform, MeCP2E1, was reported in 2004 by two different groups [14,15]. These two MeCP2 protein isoforms are identical, except for a short amino acid sequence at their N-termini, suggesting that they may share some similar and redundant functions. Both MeCP2 isoforms are nuclear proteins and commonly colocalize with the densely methylated heterochromatin foci in mouse cells [16,17]. Our team has studied the regulation and function of MeCP2 isoforms for over a decade [16,17,18,19,20,21,22,23,24]. We reported that in the brain of RTT patients carrying *MECP2* loss-of-function mutations, mTOR signaling is compromised [25]. Initiation of protein translation is a fundamental step in producing functional proteins, an important process that is also impaired in *MECP2*-deficient human RTT neurons [6]. Of note, protein translation initiation is regulated by the mTOR pathway [26]. However, the impact of MeCP2E1 and MeCP2E2 gain-of-function and/or overexpression on protein translation initiation factors has not been fully explored. Additionally, deregulation of these protein complexes in the brain of RTT patients with *MECP2* loss-of-function mutations remains largely unknown.

Here, we used lentiviral vectors to overexpress *MECP2* isoforms individually or in combination to generate human brain cells with *MECP2* gain-of-function. As control, we used a lentiviral vector with the same backbone expressing enhanced green fluorescent protein (EGFP). Overexpression of MeCP2E1 and MeCP2E2 was confirmed by Western blot (WB) and immunofluorescent (IF) experiments. Flow cytometry detection of EGFP expression in EGFP-transduced cells confirmed high transduction efficiency (>95%). Using these newly developed stably transduced cell lines, we assessed the impact of MeCP2 isoform-specific gain-of-function on MeCP2 homeostasis regulatory network (*MECP2E1/E2-BDNF*/BDNF-*miR-132*), components of mTOR-AKT signaling, ribosome biogenesis (Nucleolin and ribosomal RNAs/*rRNAs*), markers of chromatin structure (i.e., histone post-translational modifications) for euchromatin and heterochromatin, and components of the protein translation initiation machinery. We observed that combined co-transduction of both MeCP2 isoforms led to protein degradation of MeCP2E1, while MeCP2E2 was clearly detectable. Proteosome inhibition by cellular treatment with MG132 recovered MeCP2E1 protein levels within an hour, suggesting that the induced degradation of MeCP2E1 protein potentially involves MeCP2E2 through the activity of the proteosome pathway. Surprisingly, we did not detect any significant change in the components of protein translation initiation machinery as a result of MeCP2E1, MeCP2E2, or combined overexpression of both isoforms. In contrast, WB analysis of the post-mortem human RTT brain tissues with *MECP2* loss-of-function compared with controls, indicated impaired protein translation initiation, suggesting that such mechanisms may be differentially sensitive to *MECP2* gain- and loss-of-function. Taken together, our results provide further insights towards the dose-dependent functional role of MeCP2 isoforms in the brain and may shed some light into the mechanism of MeCP2-associated diseases.

## 2. Materials and Methods

### 2.1. Cell Culture Experiments, Lentiviral Production, Viral Transduction, and Flow Cytometry Assays

Daoy cells (ATCC HTB-186) were purchased from ATCC and cultured in MEM supplemented with 10% FBS, 1 mM sodium pyruvate, and 1% penicillin–streptomycin–glutamine (Gibco, Thermo Fisher Scientific, Waltham, MA, USA) as we have reported [27]. HEK-293T cells were cultured in DMEM supplemented with 10% FBS and 1% penicillin–streptomycin–glutamine as described earlier [28]. We had reported the construction of Lentiviral vectors for *MECP2* isoforms (Lenti-EF1*α*-E1 and Lenti-EF1*α*-E2), and Lenti-EF1*α*-EGFP before [28], and received them from Dr. James Ellis, The Hospital for Sick Children, Toronto, ON, Canada. Lentiviral plasmids were transformed into DH5α competent cells as previously [28], and plasmid preparation was carried out with the PureLink HiPure Plasmid Midiprep kit (Invitrogen, Thermo Fisher Scientific) according to the manufacturer’s protocol. Lentiviral particles were produced in HEK 293T cells seeded at a density of 2.3 × 10^6^ cells in T25 flasks, as reported [28]. Cells were transfected with either Lenti-EF1*α*-E1, Lenti-EF1*α*-E2, Lenti-EF1*α*-EGFP, or combination of both (Lenti-EF1*α*-E1+Lenti-EF1*α*-E2), with additional plasmids for each transfection (*REV*, *TAT*, *GAG*/*POL*, and *VSVG*), as reported [28,29]. Transfection was carried out by Lipofectamine 2000 (Thermo Fisher Scientific) as reported [28,30], and media was refreshed after 16 hours (h). Daoy cell transduction was performed with filtered viral supernatants from the HEK293T cells in the presence of 6 ug/mL polybrene. Control cells were treated with media including polybrene. Transduced cells were incubated overnight, and media was changed after 16 h. Cells were incubated for another 48 h, before being passaged, and we monitored EGFP expression by live cells fluorescence microscopy (Axio Vert.A1 inverted microscope, Carl Zeiss Microscopy, Oberkochen, Germany). Lenti-EF1*α*-EGFP transduced cells were analyzed by flow cytometry along with non-transduced controls. Flow cytometry was performed using Guava easyCyte 8HT system (Millipore, Guava Technologies, Ottawa, ON, Canada), and data were analyzed by FlowJo software as reported [28] (flow cytometry was carried out as a paid service at the Regenerative Medicine Flow Cytometry core, University of Manitoba, Winnipeg, MB, Canada).

### 2.2. Cell Viability MTT Assays and Drug (MG132) Treatment

Transduced Daoy cells with Lenti-EF1*α*-E1+Lenti-EF1α-E2 were seeded in 96-well plates at 5000 cells per well. Once cells reached to confluency, media was removed, and fresh media was added in the presence or absence of MG132 (Sigma-Aldrich, St. Louis, MO, USA). Different concentrations of MG132 at specific time points were first tested for cell viability assay by MTT reagent (Sigma-Aldrich), and MTT was carried out as reported [27,31,32,33]. For WB experiments with MG132, transduced Daoy cells with Lenti-EF1*α*-E1+Lenti-EF1*α*-E2 were cultured in 10 cm plates, and once reached to confluency, MG132 was added at 2.5 µM in fresh media. Cells were harvested at different time points after MG132 treatment, total protein was extracted from cell pellets, and WB was completed.

### 2.3. Protein Extraction and Western Blot Experiments

Total protein was extracted as reported [27,28] and was quantified by Bradford protein assay. WB was carried out as reported [34,35,36], using PVDF or Nitrocellulose membranes, with primary and secondary antibodies listed in Table 1 and Table 2, respectively.

### 2.4. Enzyme-Linked Immunosorbent Assay (ELISA)

Human BDNF ELISA kit from Sigma-Aldrich (RAB0026) was used according to the manufacturer’s instructions and as reported [23]. SpectraMax M2e plate reader (Molecular Devices, San Jose, CA, USA) and Softmax Pro 5.3 https://www.moleculardevices.com were used for measurement of absorbance and calculation of concentrations (ng/mg of total protein).

### 2.5. Immunofluorescence Experiments and Microscopic Analysis

Cells were seeded in Nunc Lab-Tek II 8-well chamber slides (Thermo Fisher Scientific) or on glass coverslips in 24-well plates, coated with 0.1% gelatin. Cells were fixed by 4% paraformaldehyde (Electron Microscopy Sciences, Hatfield, PA, USA) and stained using primary and secondary antibodies and DAPI (Calbiochem, Merck Millipore) counterstaining, as reported [16]. Imaging was performed on the Zeiss Axio Observer.Z1 (Carl-Zeiss) equipped with the AxioCam MRm. Images were acquired using Zen 2.6 Pro (Blue) software, Carl Zeiss Canada Ltd, Toronto, ON, Canada.

### 2.6. Total RNA Extraction, Nascent RNA Collection and Isolation, Real-Time Reverse-Transcriptase Polymerase Chain Reactions (RT-PCR), and MicroRNA Analysis

RNA extraction was done as reported using TRIzol (Thermo Fisher Scientific) [23,37]. Extracted RNA was treated with DNase using TURBO DNase (Ambion Life Technologies, Thermo Fisher Scientific). For microRNA analysis, total RNA was extracted by using the MagMAX *mir*Vana total RNA isolation kit (Applied Biosystems, Thermo Fisher Scientific), as per manual instructions. We synthesized the cDNA and performed quantitative RT-PCR by using the Applied Biosystems 7500 Fast Real-Time PCR System, as previously reported [22,25,38]. Reactions were composed of 25 ng template cDNA, gene-specific primers (Table 3), PowerUp SYBR Green Master Mix (Applied Biosystems, Thermo Fisher Scientific), and water. Target gene C_T_ values were normalized to the housekeeping gene *GAPDH.* Fold change values were calculated by the 2^−ΔΔCT^ as previously reported [20,22]. Table 3 provides a list of primers. The process of nascent RNA metabolic labelling of newly synthesized RNA was carried out as recently reported [22]. Nascent RNA was isolated with the Click-iT Nascent RNA Capture Kit (Invitrogen, Thermo Fisher Scientific). Cells were labelled with 0.1 mM ethynyl-uridine (EU) in the culture media for different time points, depending on the experiment. Cells were harvested, snap frozen, and RNA was extracted with the RNeasy Mini Kit or MagMAX *mir*Vana Total RNA isolation kit as described earlier [22]. The process of cDNA synthesis for the newly synthesized RNA was carried out as recently reported [22]. Quantitative RT-PCR was performed with gene-specific primers, and the resulting values were normalized to *GAPDH* as the housekeeping gene. RNA for miRNA analysis was prepared using the TRIzol or *mir*Vana RNA extraction method. Analysis of microRNAs was performed with TaqMan^TM^ MicroRNA Assays (Applied Biosystems), as reported previously [23]. RT-PCR was carried out using assay-specific 20X TaqMan MicroRNA Assay, TaqMan Universal PCR Master Mix (Applied Biosystems) on the Applied Biosystems 7500 Fast Real-Time PCR system. The following assays were used: *has-miR-132* (Assay ID 000457) and *U6 snRNA* (Assay ID 001973). Target gene *miR-132* C_T_ values were normalized to the housekeeping gene *U6,* with reporting fold change values through calculating 2^−ΔΔCT^ [23].

### 2.7. Human Brain Tissues

In this study, three female human frontal cerebrum brain tissues from RTT patients were used in parallel to age- and sex-matched non-RTT control tissues. Human brain samples were obtained from the “University of Maryland Brain and Tissue Bank, which is a Brain and Tissue Repository of the NIH Biobank (at NIH NeuroBioBank Program: neurobiobank.nih.gov)”. The completed research on human brain tissues was peer-reviewed at the University of Manitoba Research Ethical Board at the Bannatyne Campus and was approved under the REB protocol # HS20095 (H2016:337). For information on tested human brain tissues, please refer to Table 4.

### 2.8. Statistics

Statistical analysis was performed by GraphPad Prism v9.0.0 (121) https://www.graphpad.com/scientific-software/prism/, and GraphPad Prism 9 v 9.1.1 (223). Comparisons between 3 or more groups were analyzed by one-way or two-way ANOVA, followed by the Tukey multiple comparisons test, with an alpha of 0.05. Comparisons between 2 groups were analyzed by unpaired *t*-test with Welch’s correction, with an alpha of 0.05. Levels of significance were considered as *: *p* < 0.05, **: *p* < 0.01, ***: *p* < 0.001, ****: *p* < 0.0001.

## 3. Results

### 3.1. Lentiviral Expression of MeCP2 Isoforms Provided Stable and Long-Term Expression in Daoy Cells

**Figure 1:**
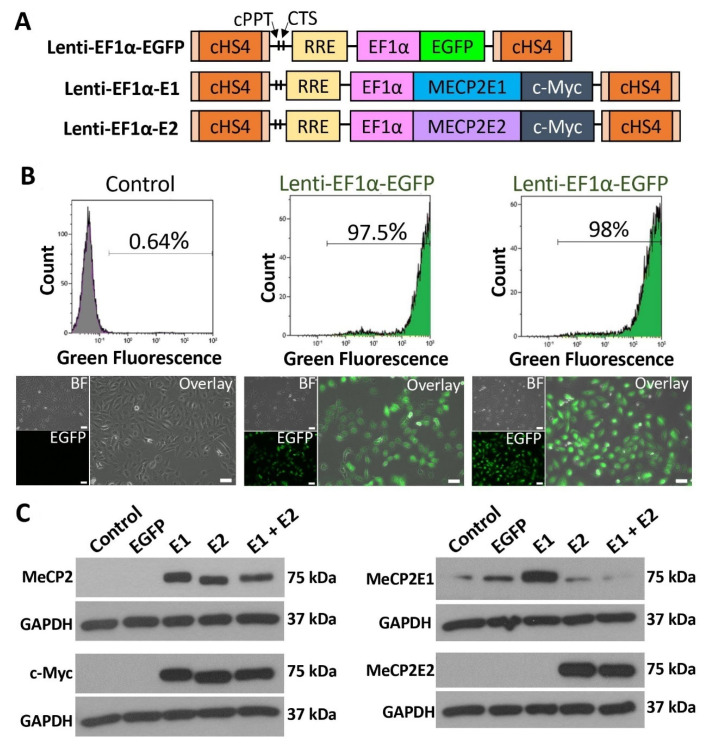
Establishment of MeCP2 isoform-specific overexpressing human Daoy brain cells using lentiviral vectors. (**A**) Schematics of lentiviral vectors used to express *EGFP*, *MECP2E1,* or *MECP2E2* under the control of the EF1*α* promoter. MeCP2 isoforms are tagged with a C-terminal c-Myc tag. cHS4: chicken *β-globin* locus hypersensitive site 4; cPPT: central poly purine tract; CTS: central terminal sequence; RRE: Rev-responsive element (Part A is adapted from Rastegar et al. 2009) [28]. (**B**) Flow cytometry analysis and fluorescence microscope images of non-transduced control and EGFP expressing cells. BF: bright field, scale bars represent 50 μm. (**C**) Western blot analysis of total MeCP2, c-Myc tag, and MeCP2E1/E2 isoforms in non-transduced control Daoy cells, EGFP-transduced (EGFP), E1-transduced (E1), E2-transduced (E2), and E1 + E2-tranduced (E1 + E2) cells.

To study the impact of isoform-specific *MECP2* gain-of-function, we generated stably transduced Daoy brain cells by using lentiviral vectors for *MECP2E1*, *MECP2E2,* and EGFP. Daoy cells are human medulloblastoma brain cells that are commonly used to study *MECP2*/MeCP2 regulation and function by independent groups, including us [22,40,41]. We used our previously developed and validated isoform-specific lentiviral vectors Lenti-EF1*α*-E1 and Lenti-EF1*α*-E2 [28]; schematics of these vectors are shown in Figure 1A. Lentiviral transduction was carried out for MeCP2E1 and MeCP2E2 individually, and in combination, being referred to E1-transduced, E2-transduced, and E1 + E2-transduced cells hereafter, respectively. Control samples included parallel transduction with an EGFP lentiviral vector with the same lentiviral backbone, along with Daoy non-transduced control cells treated with polybrene, a chemical that was used in all conditions for increasing transduction efficiency.

Daoy cells that were transduced with Lenti-EF1*α*-EGFP control vector were analyzed by flow cytometry one-week post-transduction in two independent biological replicates, with 97.5% and 98% transduction efficiency (Figure 1B, top row). EGFP was also clearly visible by fluorescence microscopy (Figure 1B, bottom row). Protein extracts of non-transduced control, EGFP-transduced, E1-transduced, E2-transduced, and E1 + E2-transduced cells were analyzed by WB. Four parallel sets of protein extracts were probed with primary antibodies for total MeCP2 (recognizing both isoforms), c-Myc (detecting the protein tag), and our reported [16,17] custom-made anti-MeCP2E1 and anti-MeCP2E2 antibodies (Figure 1C). The commercial anti-MeCP2 antibody binds to the C-terminus of MeCP2 and therefore recognizes both MeCP2E1 and MeCP2E2. This antibody detected MeCP2 at 75 kDa in E1-, E2-, and E1 + E2-transduced cells but not in non-transduced or EGFP-transduced control cells. WB with c-Myc primary antibody was carried out to confirm the presence of c-Myc tag on the overexpressed MeCP2 proteins. The c-Myc tag was detected in E1-, E2-, and E1 + E2-transduced cells at 75 kDa, the same molecular weight as total MeCP2, confirming detection of the overexpressed proteins from the lentiviral vectors. These samples were also tested for MeCP2E1 and MeCP2E2 by probing with isoform-specific antibodies developed in our lab, recognizing the unique N-terminus of each isoform [16,17]. Probing with anti-MeCP2E1-specific antibody indicated that MeCP2E1 was overexpressed in E1-transduced cells but not in E1 + E2-transduced cells (Figure 1C). We detected some level of the endogenous MeCP2E1 in non-transduced, EGFP-transduced, E2-transduced, and E1 + E2-transduced cells with clear overexpression in E1-transduced cells. Probing with anti-MeCP2E2-specific antibody, we detected MeCP2E2 in both E2-transduced and E1 + E2-transduced cells, but not in non-transduced, EGFP-transduced, and E1-transduced cells. MeCP2 isoforms were detected at approximately 75 kDa, which has been reported previously [16,17,23,28]. Detection of MeCP2 with the total MeCP2 and c-Myc antibodies showed MeCP2 running at a slightly lower molecular weight in E2-transduced cells compared with E1-transduced cells. This is expected due to the slight size difference between the two isoforms; as MeCP2E1 is 498 amino acids, and MeCP2E2 is 486 amino acids, MeCP2E1 is therefore slightly heavier and runs marginally higher than MeCP2E2. This size difference has also been detected by WB in previous studies [16,17,28].

**Figure 2:**
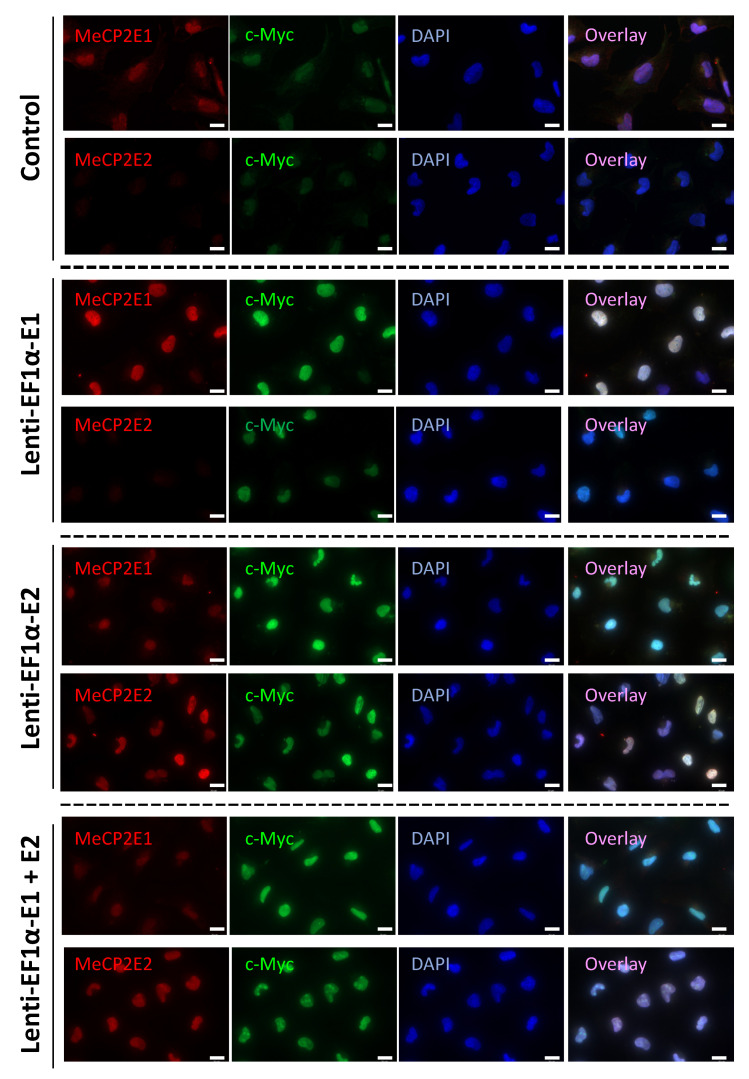
Immunofluorescence imaging of transduced Daoy cells. Control and transduced cells with indicated lentiviruses are shown. Scale bar represents 20 μm.

Next, MeCP2-overexpressing Daoy cells were subjected to immunofluorescence experiments. Cells were double-stained with three combinations of primary antibodies, total MeCP2 + c-Myc, MeCP2E1 + c-Myc, and MeCP2E2 + c-Myc. Double-staining with c-Myc antibody was performed to evaluate whether the MeCP2 antibodies detected the transduced proteins overexpressed from the lentiviral vectors. The two primary antibodies were distinguished by using secondary antibodies with different fluorophores—one conjugated to the AlexaFluor 488 and the other to AlexaFluor 594—and cells were counter-stained with DAPI to observe cellular nuclei. Total MeCP2 and c-Myc double-staining confirmed overexpression of MeCP2 from lentiviral vectors in E1-transduced, E2-transduced, and E1 + E2-transduced cells (Supplementary Figure S1). Overlay images of antibody labeling with DAPI staining indicated that overexpressed MeCP2 was localized to the nucleus. A faint level of staining with both MeCP2 and c-Myc antibodies was observed in non-transduced control cells, which could be due to the endogenous MeCP2 and c-Myc proteins that are expressed in Daoy cells (Supplementary Figure S1). Double-staining with MeCP2E1 + c-Myc antibodies in non-transduced control cells showed detection of endogenous MeCP2E1 and c-Myc (Figure 2).

E1-transduced cells showed overexpression of MeCP2E1 coupled with increased c-Myc staining (Figure 2). Endogenous MeCP2E1 was also detected in E2-transduced and E1 + E2-transduced cells along with overexpressed c-Myc due to the presence of MeCP2E2 overexpression in those cells (Figure 2). Overlay images confirmed localization of MeCP2E1 and c-Myc in the nucleus. Double-staining with MeCP2E2 + c-Myc antibodies in non-transduced cells indicated the lack of MeCP2E2 staining and faint endogenous staining of c-Myc (Figure 2). E1-transduced cells were negative for MeCP2E2 but had elevated staining for c-Myc due to overexpression of MeCP2E1 from lentiviral transduction (Figure 2). E2-transduced and E1 + E2-transduced cells both showed strong staining for MeCP2E2 and c-Myc (Figure 2). Our immunofluorescence results confirm that Daoy transduced cells maintained stable long-term overexpression of MeCP2 isoforms, as IF results are from transduced cells that have been in culture for ~25 passages. Taken together, these results confirm that MeCP2 overexpression in Daoy cells were in the proper sub-cellular localization (nucleus) and that these cells maintained long term transduction of these proteins.

### 3.2. Cell Viability Assays Show Time-Dependent Survival Inhibition in MeCP2E2 Overexpressing Cells Compared with Non-Transduced Daoy Cells

Previous studies have shown that overexpression of MeCP2E2 promotes apoptosis in rodent brain cells [42]. Thus, we tested the cell viability of MeCP2E1 and E2 overexpressing Daoy cells by MTT assay. This assay determines cell viability by adding methyl-thiazolyl-tetrazolium (MTT) to cultured cells and measuring by spectrophotometry the amount of MTT-formazan produced. MTT assay was performed after the cells had been passaged about 10 times since the start of the transductions. Non-transduced control, EGFP-transduced, E1-transduced, E2-transduced, and E1 + E2-transduced Daoy cells were seeded at equal densities (~2000 cells) in 96-well plates, and MTT assay was performed after 24 h, 48 h, and 72 h. Comparisons between the five cell groups at each time point are shown in Supplementary Figure S2A. Statistical significance was assessed by two-way ANOVA followed by Tukey’s multiple comparisons tests. Results of two-way ANOVA are shown in Supplementary Table S1. At the 24 h time point, the percentage viability of E1-, E2-, and E1 + E2-transduced cells was significantly reduced by approximately 20% (*p* < 0.05), 30% (*p* < 0.0001), and 25% (*p* < 0.001), respectively, compared with non-transduced control cells. EGFP-transduced cells did not show any significant differences compared with control cells at any time point. Only E2-transduced cells were also significantly reduced compared with EGFP-transduced cells (*p* < 0.05). EGFP-transduced cells showed a higher degree of variability, making it difficult to conclude whether the decreases in cell viability of E1- and E1 + E2-transduced cells were due specifically to MeCP2 overexpression and not an off-target effect of transduction. At the 48 h and 72 h time points, there were no statistically significant differences in the percentage cell viability in any groups of transduced cells compared with controls. MTT assay data were also analyzed by Tukey’s multiple comparisons test for differences in percentage viability between the three time points (Supplementary Figure S2B,C). Non-transduced control cell viabilities were expressed relative to the average non-transduced control cells value and therefore appeared at about 100% cell viability at each time point. EGFP-transduced cells did not show any change in their cellular viability from 24 h to 48 h to 72 h (Supplementary Figure S2B). However, E1-, E2-, and E1 + E2-transduced cells showed significant increases in percentage viability over time (Supplementary Figure S2C). E1-transduced cells showed significant increases in percentage cell survival from approximately 80% at 24 h to 100% at 72 h (*p* < 0.01), and from approximately 85% at 48 h to 100% at 72 h (*p* < 0.05) (Supplementary Figure S2C). E2-transduced cells showed increases in percentage survival from approximately 70% at 24 h to 85% at 48 h to 100% at 72 h (Supplementary Figure S2C). The differences between all the time points were statistically significant between 24 h and 48 h (*p* < 0.05) and between 24 h and 72 h (*p* < 0.0001), as well as between 48 h and 72 h (*p* < 0.05). E1 + E2-transduced cells also had increased percentage cell survival between all the time points, from approximately 75% at 24 h to 90% at 48 h and 110% at 72 h (Supplementary Figure S2C).

Taken together, our MTT results indicated that while all conditions had identical initial cell densities, percentage viabilities of E1-, E2-, and E1 + E2-transduced cells were significantly reduced compared with non-transduced controls at 24 h. However, this reduction was only significant for E2-transduced compared with EGFP-transduced cells, although EGFP-transduced cells showed no difference from non-transduced control cells. Differences in percentage cell viability specific to MeCP2-overexpressing cells became clearer when making comparisons between the different time points. EGFP-transduced cells showed no significant change in cell viability over time, but MeCP2-overexpressing cells displayed significant changes in percentage cell viability over 72 h. This indicated that the cell viability of MeCP2-overexpressing cells experiences significant change in the time following seeding, with fewer cells present at 24 h, but cell numbers reach to a similar number by 72 h. Greater and more significant changes were seen in E2-, and E1 + E2-transduced cells, indicating that the MeCP2E2 isoform may have higher impact on the viability of these cells, since E1 + E2-transduced also primarily overexpressed the E2 isoform. Further studies are required to address whether a possible increase in cell death occurs by apoptosis or other pathways related to cell death.

### 3.3. MECP2E1-E2 Homeostasis at the Transcriptional and Post-Transcriptional Levels

Our previous studies had suggested a potential negative autoregulation of MeCP2 isoforms, where we detected a reciprocal transcript expression of *Mecp2* isoforms during neural stem cell differentiation [21]. We also noticed that when the Daoy cells were transduced with both *MECP2E1* and *MECP2E2* lentiviral vectors (E1 + E2-transduced cells), we could only detect the transduced MeCP2E2 (Figure 1C).

**Figure 3:**
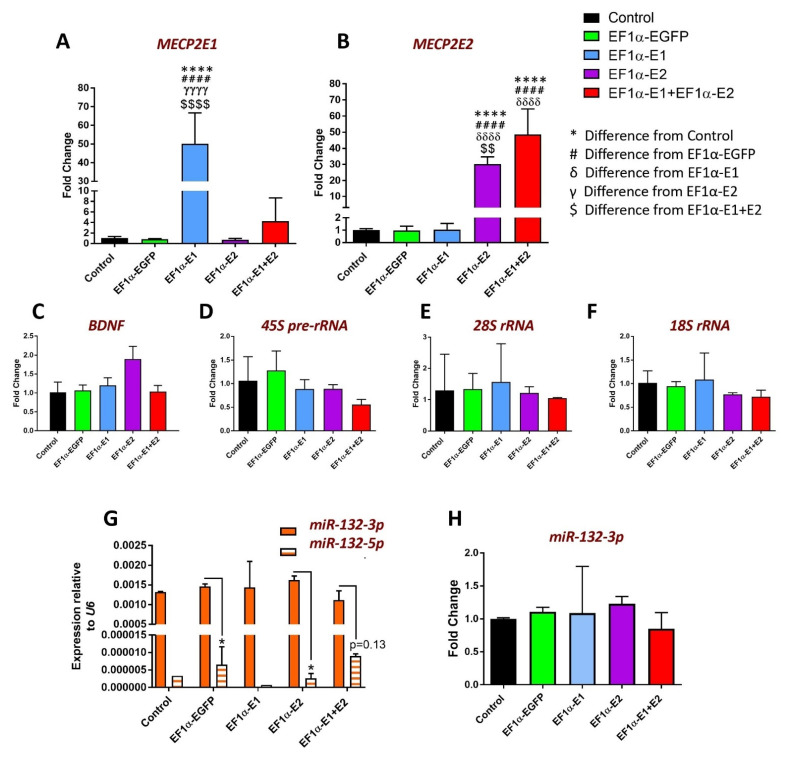
RT-PCR analysis of indicated transcripts in Daoy cells transduced with lentiviral vectors expressing MeCP2 isoforms. C_T_ values for (**A**) *MECP2E1*, (**B**) *MECP2E2*, (**C**) *BDNF*, (**D**) *45S-pre-rRNA*, (**E**) *28S rRNA*, (**F**) *18S rRNA* were normalized to the housekeeping gene *GAPDH*. Fold changes were determined relative to average non-transduced Daoy cell controls. Fold change values were analyzed by one-way ANOVA followed by Tukey’s multiple comparisons test. *n* = 5, and data are reported as mean ± SEM; *: *p* < 0.05, **: *p* < 0.01, and ****: *p* < 0.0001 (* indicates the difference from control; # indicates the difference from EF1*α*-EGFP; δ indicates the difference from EF1*α*-E1; γ indicates the difference from EF1*α*-E2; $ indicates the difference from EF1*α*-E1 + E2, respectively). The *p* = 0.13 value in (**G**) is higher than the statistical significance of * *p* < 0.05. For (**G**,**H**), normalization has been carried out with *U6* microRNA.

Hence, we decided to investigate the expression levels of *MECP2E1* and *E2* transcripts. Thus, total RNA from five sets of non-transduced, EGFP-, E1-, E2-, and E1 + E2-transduced cells were subjected to cDNA synthesis and real-time RT-PCR. Such transcript analysis would detect additive expression of the endogenous *MECP2* gene and the isoform-specific lentiviral vectors. We observed an increase in the transcript levels of *MECP2E1* by approximately 50-fold in E1-transduced cells relative to endogenous transcript levels in non-transduced controls, which was also significantly greater than EGFP-, E2-, and E1 + E2-transduced cells (*p* < 0.0001) (Figure 3A).

E1 + E2 cells showed an approximately 4-fold increase in *E1* transcripts relative to non-transduced cells, but the difference was not statistically significant compared with non-transduced, EGFP-, or E2-transduced cells, most likely due to the high variability in results for E1 + E2 cells (Figure 3A). Transcript levels of *MECP2E2* were increased by approximately 30-fold in E2-transduced cells relative to the endogenous transcripts in non-transduced controls, and there was also a statistically significant increase compared with EGFP- and E1-transduced cells (*p* < 0.0001) (Figure 3B). E1 + E2 cells showed an approximately 48-fold increase in *MECP2E2* transcripts relative to non-transduced Daoy cells, an increase which was statistically significant compared with EGFP- and E1-transduced cells. (*p* < 0.0001) (Figure 3B). The difference in *MECP2E2* transcripts between E2 and E1 + E2 cells was 1.6-fold (*p* < 0.01). In conclusion, *MECP2E1* transcripts were significantly overexpressed in E1-transduced cells but not in E1 + E2-transduced cells, while *MECP2E2* transcripts were significantly overexpressed not only in E2-transduced but also in E1 + E2-transduced Daoy cells. Transcripts expressed from the lentiviral vectors lack the 3′ UTR of *MECP2,* which is known to be post-transcriptionally regulated, for example, by microRNAs.

**Figure 4:**
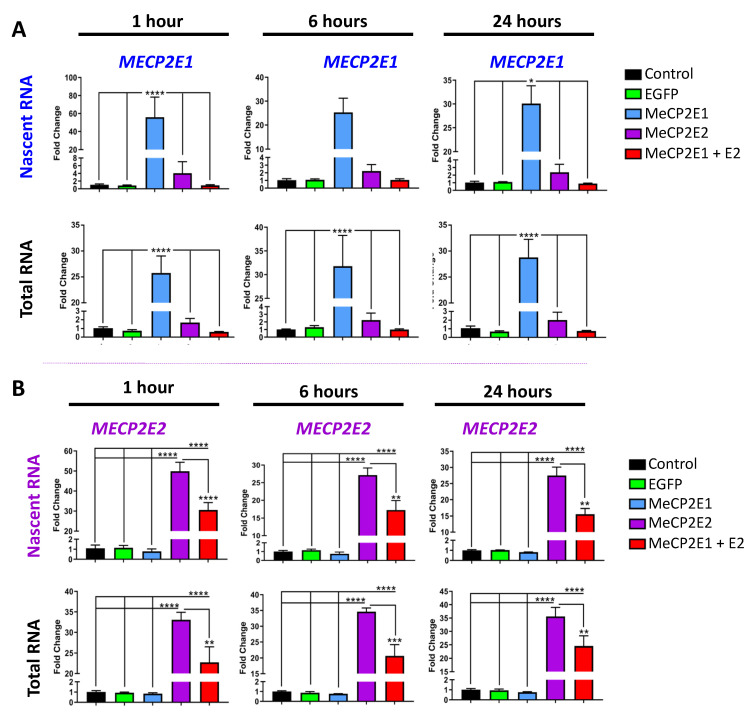
Expression of nascent and steady-state *MECP2E1* and *MECP2E2* transcripts in Daoy cells transduced with lentiviral vectors expressing MeCP2 isoforms. Nascent RNA was prepared from cells labelled with EU for 1 h, 6 h, and 24 h. Total RNA was prepared from unlabeled cells collected at the same time points. Gene expression was analyzed by RT-PCR for (**A**) *MECP2E1* transcripts and (**B**) *MECP2E2* transcripts. C_T_ values were normalized to the housekeeping gene *GAPDH,* and fold change was determined relative to average non-transduced control cells with *n* = 3, and data are reported as mean ± SEM. Fold change values were analyzed by two-way ANOVA followed by Tukey’s multiple comparisons test. The levels of significance are *: *p* < 0.05, **: *p* < 0.01, ***: *p* < 0.001, and ****: *p* < 0.0001.

Next, we studied the expression of newly synthesized nascent transcripts for both *MECP2* isoforms. Non-transduced, EGFP-, E1-, E2-, and E1 + E2-transduced cells were seeded at 50,000 cells per well of 6-well plates. After 16 h, ethynyl-uridine (EU) was added to label nascent RNA for 1 h, 6 h, and 24 h. One group of samples was labelled with EU, and a second group was left unlabeled for collection of total steady-state RNA. At each time point, three independent experimental replicates of EU-labelled and three independent replicates of unlabeled cells were analyzed. Next, we analyzed the nascent and steady-state *MECP2E1/E2* transcripts to determine the level of *MECP2* transcripts in these samples (Figure 4). The results of the two-way ANOVA are shown in Supplementary Table S2. *MECP2E1* nascent transcripts were increased compared with controls by approximately 55-fold at 1 h *p* < 0.0001), 25-fold at 6 h, (ns, *p* = 0.06), and 30-fold at 24 h (*p* < 0.05 (Figure 4A). *MECP2E1* steady-state transcripts were increased compared with controls by approximately 25-fold at 1 h (*p* < 0.0001), 30-fold at 6 h (*p* < 0.0001), and 30-fold at 24 h (*p* < 0.0001). Compared with controls, no statistically significant increase was detected for *MECP2E1* nascent and steady-state transcripts in E2- or E1 + E2-transduced cells. The levels of *MECP2E1* in E2- and E1 + E2-transduced cells were also statistically significantly different from E1-transduced cells, with identical *p*-values seen in comparison with controls. Furthermore, *MECP2E2* nascent and steady-state transcripts were significantly increased in E2- and E1 + E2-transduced cells compared with the controls (Figure 4B). *MECP2E2* nascent transcripts were increased in E2-transduced cells compared with controls by approximately 50-fold at 1 h (*p* < 0.0001), 27-fold at 6 h (*p* < 0.0001), and 27-fold at 24 h (*p* < 0.0001). *MECP2E2* nascent transcripts were increased in E1 + E2-transduced cells compared with controls by approximately 30-fold at 1 h (*p* < 0.0001), 17-fold at 6 h (*p* < 0.0001), and 15-fold at 24 h (*p* < 0.0001). The level of *MECP2E2* nascent transcripts in E2-transduced cells was approximately 1.5-fold greater than E1 + E2-transduced cells at all time points, which was statistically significant (*p* < 0.0001 at 1 h; *p* < 0.01 at 6 h and 24 h). *MECP2E2* steady-state transcripts were increased in E2-transduced cells compared with controls by approximately 33-fold at 1 h (*p* < 0.0001), 35-fold at 6 h (*p* < 0.0001), and 35-fold at 24 h (*p* < 0.0001). *MECP2E2* steady-state transcripts were increased in E1 + E2-transduced cells compared with controls by approximately 23-fold at 1 h (*p* < 0.0001), 20-fold at 6 h (*p* < 0.0001), and 25-fold at 24 h (*p* < 0.0001). The level of *MECP2E2* steady-state transcripts in E2-transduced cells was approximately 1.5-fold greater than E1 + E2-transduced cells at all time points, which was statistically significant (*p* < 0.01 at 1 h and 24 h; *p* < 0.001 at 6 h). Results of two-way ANOVA are shown in Supplementary Table S3.

**Figure 5:**
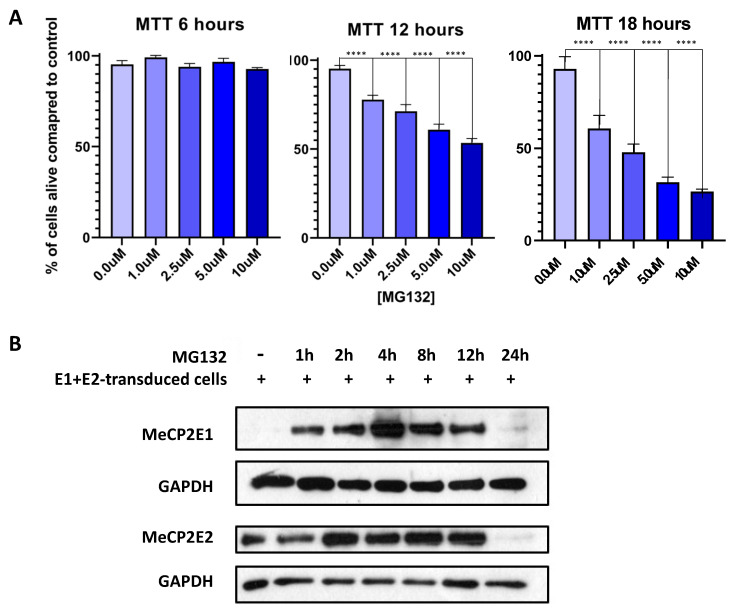
MTT cell survival assay and Western blot analysis of the E1 + E2-transduced cells treated with MG132. (**A**) Cell survival MTT assays were performed at 3 time points of 6 h, 12 h, and 18 h to determine the cell death/cell survivability with various incubation times and MG132 concentrations. All conditions were performed in *n* = 5, and data are reported as mean ± SEM with two-way ANOVA analysis, followed by Dunnett’s multiple comparisons test with significances of ****: *p* < 0.0001. (**B**) Western blot analysis of the E1 + E2-transduced Daoy cells treated with 2.5 µM of MG132, using anti-MeCP2E1 and anti-MeCP2E2 (h: hour). GAPDH loading control is shown as a housekeeping protein.

Our results indicated that *MECP2E1* transcripts were significantly increased in E1-transduced cells and that *MEPC2E2* transcripts were significantly increased in E2- and E1 + E2-transduced cells, although to a greater degree in E2-transduced cells. This in part could explain why no MeCP2E1 was detected in E1 + E2-transduced cells, but MeCP2E2 was present at the protein level. However, it will not exclude the possibility of additional regulatory mechanisms at the protein level. Hence, we investigated the possibility of proteosome pathway involvement in degrading MeCP2E1 protein. First, Daoy cells were subjected to MTT cell survival assays for different concentrations of MG132, a chemical compound that is commonly used for blocking the proteosome pathway protein degradation (Figure 5A). MTT assays were completed at 6 h, 12 h, and 18 h after MG132 was added to the culture media. Results of two-way ANOVA are shown in Supplementary Table S4. At 6 h, no change in cell survival was detected with any of the MG132 concentrations that were used (0 µM, 1 µM, 2.5 µM, 5 µM, and 10 µM). However, after 12 h of MG132 treatment, significant cell death was observed in cells that were treated with MG132, irrespective of the concentration. At 18 h time point, this reached to almost 50% cell survival in cells treated with 2.5 µM MG132. Thus, we selected this MG132 concentration to treat the E1 + E2 cells, and protein samples were collected at different time points after drug treatment (1 h, 2 h, 4 h, 8 h, 12 h, and 24 h). Collected protein samples were subjected to WB analysis with anti-MeCP2E1 and anti-MeCP2E2 antibodies.

The results showed a robust detection of MeCP2E1 within an hour following MG132 treatments, peaking around 4 h. As expected, MeCP2E2 was detectable when no MG132 was added, yet MeCP2E2 levels also increased within 2 h of MG132 treatment. Both isoforms were visibly detected up to 12 h but were undetectable by 24 h (Figure 5B). MG132 is a potent and toxic chemical for the cells, and significant induction of cell death is expected over time, and according to the MTT results (Figure 5A), at 24 h we expect cell death to occur in over 50% of cells. Taken together, these results support the presence of a post-translational degradation of the MeCP2E1 at the protein level, through regulatory mechanisms that might be mediated in part by MeCP2E2. Overall, these results support the existence of autoregulatory mechanisms for MeCP2 isoforms that may exist at multiple levels; however, the full extent of such autoregulation needs further investigations that were beyond the scope of this study.

### 3.4. Nascent RNA Analysis Identifies MeCP2 Isoform-Specific Common and Unique Target Genes

MeCP2 isoforms share all of their functional protein domains, except for their short amino acid sequence in their N-terminal regions. Thus, we investigated the role of individual MeCP2 isoforms in regulating *BDNF* and *miR-132* as known elements of the MeCP2-BDNF-*miR-132* homeostasis network. As shown in Figure 3C, no significant difference was detected for *BDNF* transcripts in E1-, E2-, or E1 + E2-transduced cells compared with non-transduced or EGFP-transduced control cells (Figure 3C). Next, we analyzed *miR-132* expression for both strands of *miR-132:* the *3p* and *5p*. These two *miR-132* strands are generated following cleavage of the *pre-miRNA* hairpin, with one strand retaining the original 3-primed end and the other the original 5-primed end. To compare expression of the two strands in non-transduced and transduced Daoy cells, the C_T_ values for each strand were normalized to *U6* and then expressed as 2^−ΔCT^ (Figure 3G). This indicated that the *3p* strand is the dominant strand expressed in Daoy cells, at levels about 300-fold higher than the *5p* strand. In non-transduced control and E1-transduced cells, only one of the four technical replicates amplified the *5p* strand, suggesting an expression level that is below the detection level. The difference between the expression of *3p* and *5p* strands was statistically significant in EGFP-transduced and E2-transduced cells (*p* < 0.05). The *p*-value for the comparison of *5p* and *3p* transcripts in E1 + E2-transduced cells was not significant (0.13). These results agree with our published findings on the levels of *miR-132-3p* and *-5p* strands in the human brain tissues which also indicated *3p* is the predominant strand [23]. When the fold changes in *3p* transcripts were determined relative to non-transduced cells, there were no significant differences in any of the transduced cells (Figure 3H).

Our results indicated that *BDNF* transcripts show a trend of increased transcript levels in E2-transduced cells that were not statistically significant. However, it is possible to obtain clearer results if we specifically study the level of nascent RNA. Thus, we analyzed the nascent and total steady-state RNA levels, with uniform seeding and collection time points between samples, as was described and presented for *MECP2* isoforms in Figure 4.

**Figure 6:**
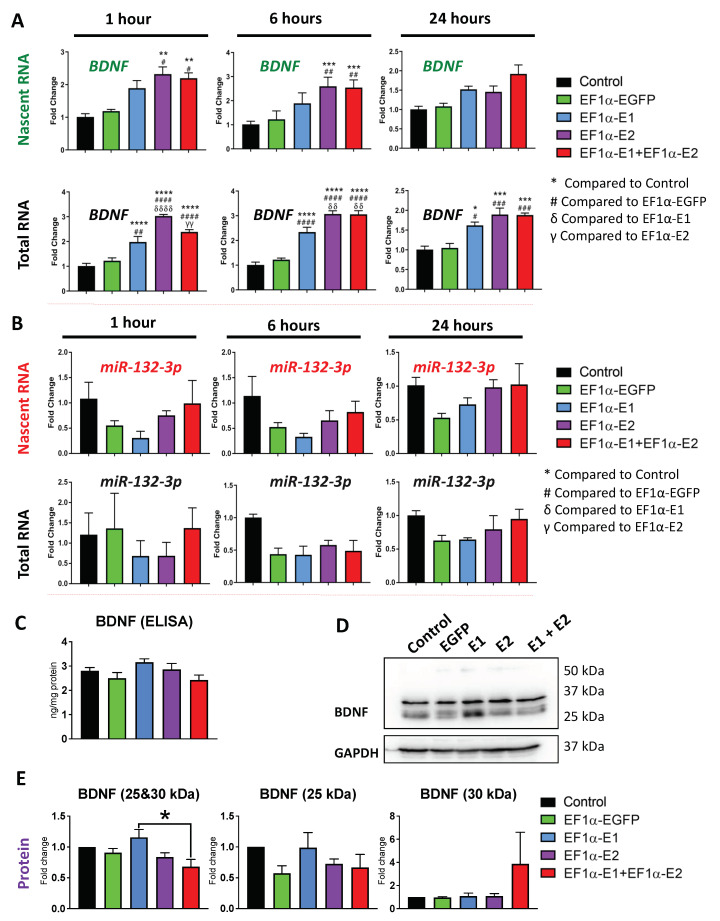
Expression of nascent and steady-state *BDNF* and *miR-132-3p* transcripts, and BDNF protein in Daoy cells transduced with lentiviral vectors expressing MeCP2 isoforms. Nascent RNA was prepared from cells labelled with EU for 1 h, 6 h, and 24 h. Total RNA was prepared from unlabeled cells collected at the same time points. Gene expression was analyzed by RT-PCR for (**A**) *BDNF* transcripts, with GAPDH as the housekeeping gene, and (**B**) *miR-132-3p* transcripts with microRNA *U6* as the housekeeping control. For A and B, the C_T_ values were normalized to the housekeeping gene, and fold change was determined relative to average non-transduced control cells. *n* = 3, and data are reported as mean ± SEM. Fold change values were analyzed by two-way ANOVA followed by Tukey’s multiple comparisons test, *: *p* < 0.05, **: *p* < 0.01, ***: *p* < 0.001, and ****: *p* < 0.0001 (* indicates the difference from control; # indicates the difference from EF1*α*-EGFP; δ indicates the difference from EF1*α*-E1; γ indicates the difference from EF1*α*-E2, respectively). (**C**) ELISA experiments for BDNF protein in analyzed total cell extracts from non-transduced Daoy cell controls and transduced cells with different lentiviral vectors, with *n* = 3, and data reported as mean ± SEM. (**D**) Western blot protein analysis of BDNF in collected protein extracts of non-transduced Daoy cells and transduced cells with different lentiviral vectors. (**E**) Quantification of BDNF signals detected by Western blot in D, with *n* = 4, and data reported as mean ± SEM.

We detected significant increases in *BDNF* nascent and steady-state transcripts (Figure 6A). After 1 h of EU-labeling, nascent *BDNF* transcripts were increased by approximately 2.3-fold in E2-transduced cells (*p* < 0.01) and 2.2-fold in E1 + E2-transduced cells (*p* < 0.01) compared with non-transduced control cells. E2- and E1 + E2-transduced cells were also significantly increased compared with EGFP-transduced control cells (*p* < 0.05). After 6 h of EU-labeling, the fold changes in E2- and E1 + E2-transduced cells were increased to 2.6-fold and 2.5-fold, respectively, and were significantly different compared with non-transduced control cells (*p* < 0.001) and EGFP-transduced control cells (*p* < 0.01). Following 24 h of EU-labeling, the nascent *BDNF* transcripts were not statistically different in transduced cells compared with controls.

Steady-state *BDNF* transcripts were significantly increased in E1-, E2-, and E1 + E2-transduced cells at 1 h, 6 h, and 24 h compared with both non-transduced and EGFP-transduced controls. At the 1 h time point, *BDNF* transcripts were increased relative to non-transduced control cells in E1-, E2-, and E1 + E2-transduced cells by approximately 2-fold (*p* < 0.0001), 3-fold (*p* < 0.0001), and 2.4-fold (*p* < 0.0001), respectively. The differences were also statistically significant compared with EGFP-transduced controls (*p* < 0.01, *p* < 0.0001, and *p* < 0.0001, respectively). At the 6 h time point, *BDNF* transcripts were increased relative to non-transduced control cells in E1-, E2-, and E1 + E2-transduced cells by approximately 2.3-fold (*p* < 0.0001), 3-fold (*p* < 0.0001), and 3-fold (*p* < 0.0001), respectively. These differences were also statistically significant compared with EGFP-transduced controls (*p* < 0.0001 for all *MECP2*-overexpressing cells). At the 24 h time point, the fold changes were slightly lower, but *BDNF* transcripts were still increased relative to non-transduced control cells in E1-, E2, and E1 + E2-transduced cells by approximately 1.6-fold (*p* < 0.05), 1.9-fold (*p* < 0.001), and 1.9-fold (*p* < 0.001), respectively. The differences were statistically significant compared with EGFP-transduced controls (*p* < 0.05, *p* < 0.001, and *p* < 0.001, respectively). There were also statistically significant differences in steady-state *BDNF* transcripts between the E1-, E2-, and E1 + E2-transduced cells at 1 h and 6 h time points. At the 1 h time point, the *BDNF* levels in E2-transduced cells were significantly higher than E1-transduced cells by about 1.5-fold (*p* < 0.0001) and E1 + E2-transduced cells by about 1.25-fold (*p* < 0.01). At the 6 h time point, the *BDNF* levels in E2- and E1 + E2-transduced cells were both significantly higher compared with E1-transduced cells by about 1.3-fold (*p* < 0.01). The greater degree of fold-changes and statistical significance in steady-state *BDNF* compared with nascent *BDNF* may be due to the fact that the nascent RNA samples represent a smaller fraction of transcripts, being only those newly synthesized transcripts produced during the time frame of labeling. The steady-state samples contain a greater number of transcripts, also representing the transcripts present before the EU-labeling was begun. These gene expression results indicate that overexpression of either MeCP2E1 or E2 isoforms resulted in increases in nascent *BDNF* transcription.

Next, we analyzed the effect of MeCP2 overexpression on nascent *miR-132* expression. Nascent and steady-state *miRNA* samples were prepared similarly to the other gene targets except TaqMan microRNA assays were used. The *miR-132-3p* strand was analyzed as it was determined to be the predominant strand expressed in Daoy cells. Nascent *miR-132-3p* transcripts showed no statistically significant changes (Figure 6B), but some variation was observed in the transduced cells compared with non-transduced control cells, including in EGFP-transduced cells in which *miR-132-3p* was reduced about 0.5-fold at every time point. Results of two-way ANOVA analysis indicate that transduction had a significant impact overall (F = 4.386, *p* < 0.01) (Supplementary Table S2). This indicates that an off-target effect of transduction may impact *miR-132-3p* levels. This trend was also observed for steady-state *miR-132-3p* transcripts, though not statistically significant by two-way ANOVA. Therefore, it appeared that MeCP2 overexpression in Daoy cells did not have any significant effects on *miR-132* that were specific to MeCP2 overexpression in individual overexpressing cell line(s). Among MeCP2 target genes are fundamental ubiquitous genes such as *Nucleolin* and ribosomal RNA (*rRNA*) genes [25].

**Figure 7:**
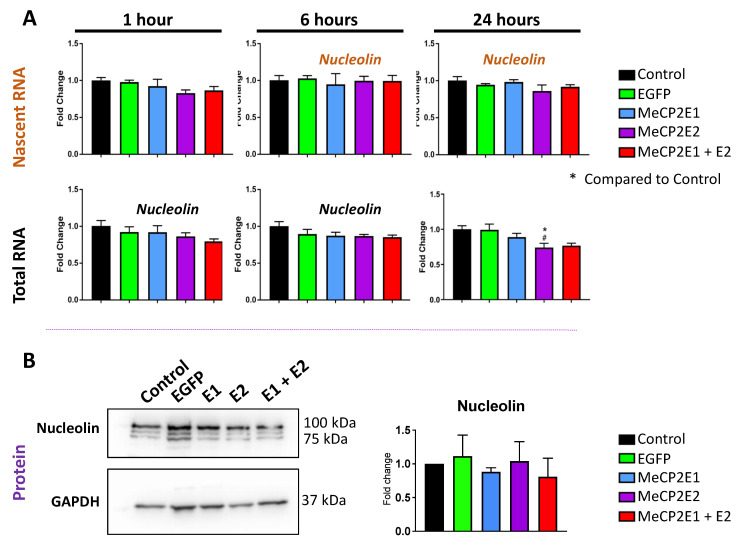
Expression of the nascent and steady-state *Nucleolin* transcripts and Nucleolin protein in Daoy cells transduced with lentiviral vectors expressing MeCP2 isoforms. (**A**) Nascent RNA was prepared from cells labelled with EU for 1 h, 6 h, and 24 h. Total RNA was also prepared from unlabeled cells collected at the same time points. Gene expression was analyzed by RT-PCR for *Nucleolin*. C_T_ values were normalized to the housekeeping gene *GAPDH,* and fold change was determined relative to average non-transduced control cells. *n* = 3, and data are reported as mean ± SEM. Fold change values were analyzed by two-way ANOVA followed by Tukey’s multiple comparisons test, *: *p* < 0.05 (* indicates the difference from control; # indicates the difference from EF1*α*-EGFP, respectively). (**B**) Western blot protein analysis of Nucleolin in collected protein extracts of non-transduced Daoy cells and transduced cells with different lentiviral vectors and their quantification, with *n* = 6, and data reported as mean ± SEM.

Real-time qRT-PCR was completed for analyzing the expression levels of *Nucleolin, 45S pre-rRNA, 28S rRNA,* and *18S rRNA. Nucleolin* transcripts were unchanged at the nascent and steady-state levels except for a slight significant decrease in steady-state levels in E2-transduced cells at 24 h compared with non-transduced and EGFP-transduced cells (*p* < 0.05) (Figure 7A). These results indicate that *Nucleolin* transcription is not impacted by MeCP2 overexpression in Daoy cells.

**Figure 8:**
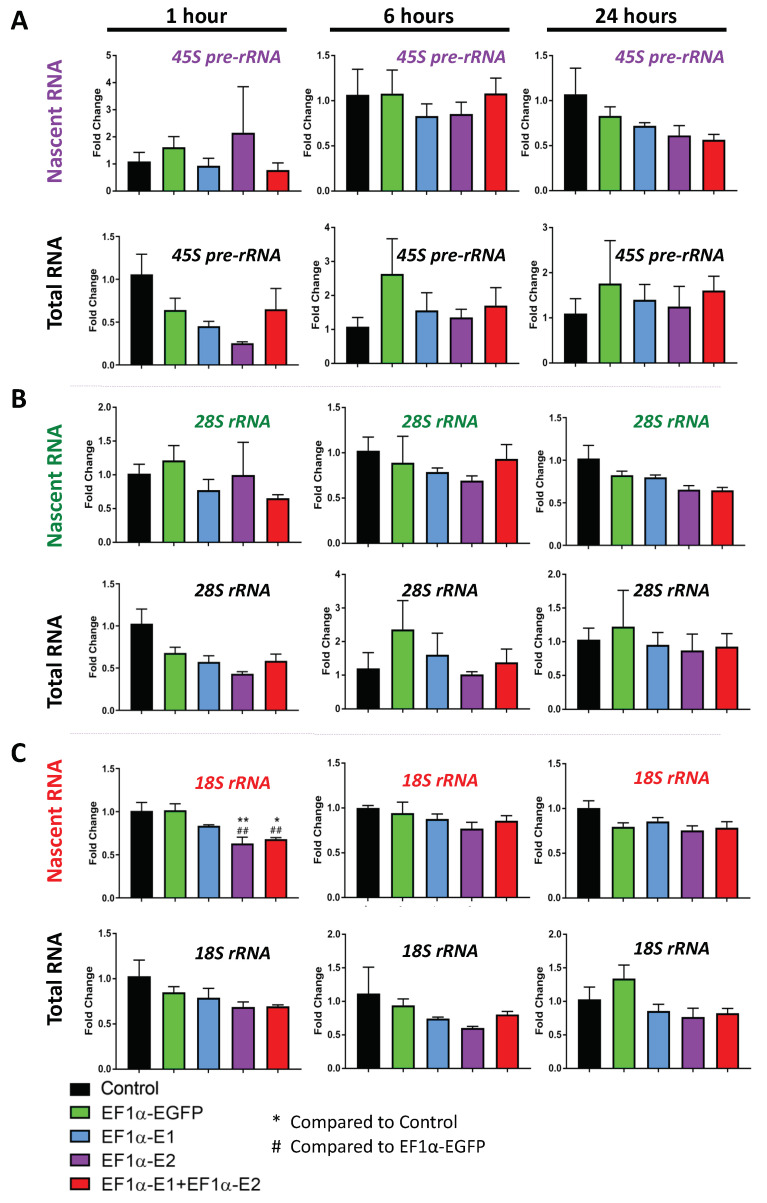
Expression of the nascent and steady-state *45S pre-rRNA* and *28S* and *18S rRNA* transcripts in Daoy cells transduced with lentiviral vectors expressing MeCP2 isoforms. Nascent RNA was prepared from cells labelled with EU for 1 h, 6 h, and 24 h. Total RNA was prepared from unlabeled cells collected at the same time points. Gene expression was analyzed by RT-PCR for (**A**) *45S pre-rRNA* transcripts, (**B**) *28S rRNA* transcripts, and (**C**) 18S *rRNA* transcripts. C_T_ values were normalized to the housekeeping gene *GAPDH,* and fold change was determined relative to average non-transduced control cells. *n* = 3, and data are reported as mean ± SEM. Fold change values were analyzed by two-way ANOVA followed by Tukey’s multiple comparisons test: *: *p* < 0.05 and **: *p* < 0.01 (* indicates the difference from control; # indicates the difference from EF1*α*-EGFP, respectively).

Analysis of the *45S pre-rRNA* transcripts resulted in quite high variability between replicates in some samples, and no statistically significant differences were observed in nascent or steady-state transcripts (Figure 8A). The *28S rRNA* transcripts also showed no statistically significant differences (Figure 8B). At 1 h, the *18S rRNA* nascent transcripts were significantly reduced in E2- and E1 + E2-transduced cells, reaching to approximately 0.6-fold and 0.7-fold, respectively, which were both statistically significant compared with both non-transduced (*p* < 0.01 and *p* < 0.05, respectively) and EGFP-transduced controls (*p* < 0.01) (Figure 8C). Two-way ANOVA analysis indicated no significant effects overall by transduction or time for nascent *45S pre-rRNA* and *28 rRNA*; however, a significant effect by transduction was detected for nascent *18S rRNA* (F = 8.905, *p* < 0.0001) (Supplementary Table S2). Although steady-state *45S pre-rRNA*, *28S rRNA,* and *18S rRNA* transcripts showed no significant differences by multiple comparisons, the two-way ANOVA test indicated a significant effect by time for *45S pre-rRNA* (F = 6.585, *p* < 0.01) and *28S rRNA* (F = 6.868, *p* < 0.01), indicating that the variation detected over time points is significant (Supplementary Table S3). There was also a significant effect by transduction for steady-state *18S rRNA* (F = 3.992, *p* < 0.05); however, none of the individual comparisons were statistically significant (Supplementary Table S3). The only clear effect specific to MeCP2 was the decrease in nascent *18S rRNA* transcripts at the 1 h time point.

Next, we studied the protein level of BDNF and Nucleolin. Surprisingly, the transcriptional induction of *BDNF* by *MECP2* overexpression did not lead to increased BDNF protein levels studied by WB and ELISA (Figure 6C–E). Similar results were also detected for Nucleolin protein, where no change was detected under any transduction conditions (Figure 7B). Results of one-way ANOVA are shown in Supplementary Table S5. This indicates that Daoy cells may have additional regulatory compensatory mechanisms in place to control the BDNF protein levels.

### 3.5. Components of the mTOR Pathway Show Differential Sensitivity to MECP2 Gain- and Loss-of-Function

**Figure 9:**
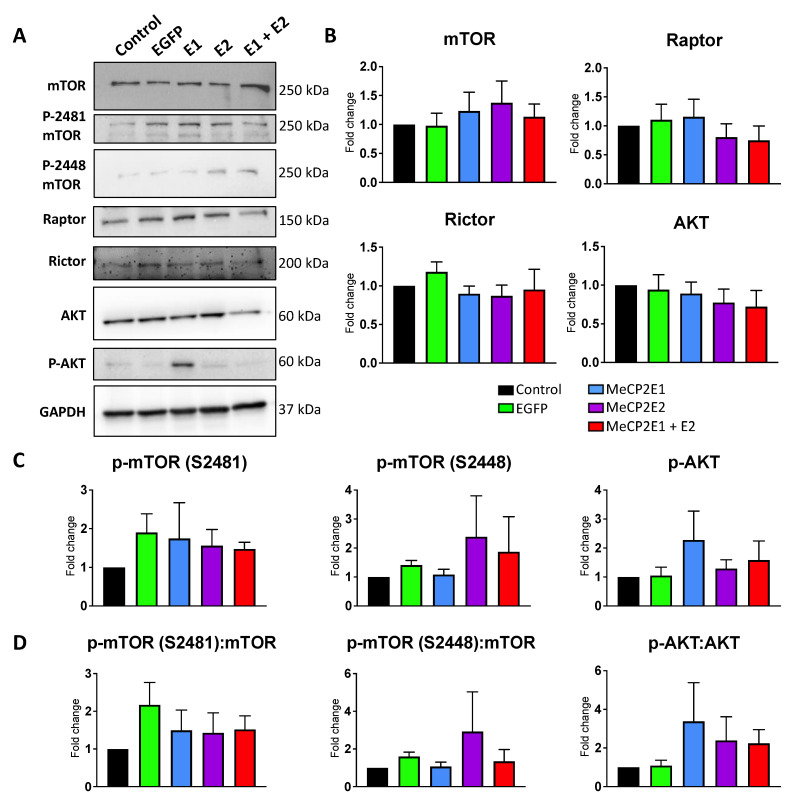
Protein analysis of components of the mTOR and AKT pathways in Daoy cells transduced with lentiviral vectors overexpressing MeCP2 isoforms. (**A**) Western blot analysis of the indicated proteins in non-transduced control Daoy cells, EGFP-transduced (EGFP), E1-transduced (E1), E2-transduced (E2), and E1 + E2-tranduced (E1 + E2) cells. (**B**) Quantification of the indicated pan total proteins from A. (**C**) Quantification of the indicated phosphorylated proteins from A. (**D**) Ratio analysis of the phosphorylated proteins from A versus their own non-phosphorylated pan total proteins. *n* = 4–6 (*n* = 4 for mTOR, p-mTOR (S2481), p-mTOR (S2448), and Rictor; *n* = 5 for p-AKT, *n* = 6 for AKT and Raptor), and data are reported as mean ± SEM. For ratio of phosphorylated proteins to their pan protein abundances (Part **D**), samples from the same biological replicate were analyzed against the pan proteins from the same set. For the reported ratios, where *n* is different for the phosphorylated proteins versus their pan protein, *n* for the ratio would be equal to the lowest *n* within the phospho-pan protein pair.

We previously reported that *MECP2* loss-of-function mutations are associated with deregulation of the mTOR pathway in the cerebellum of RTT patients [25]. The cerebellum is a specific region of the brain that is involved in locomotor function with a high density of neurons [43]. In order to study the impact of *MECP2* gain-of-function in the cerebellum cells, we benefited from our newly developed *MECP2E1* and *MECP2E2* gain-of-function Daoy cells. Thus, collected cell extracts from the non-transduced Daoy cells along with the EGFP-, E1-, E2-, and E1 + E2-transduced cells were subjected to WB analysis for mTORC1 and mTORC2 (mTOR, phospho-S2448-mTOR, phospho-S2481-mTOR, Raptor, and Rictor), along with AKT and phospho-AKT (p-AKT). Compared with non-transduced control cells and EGFP-transduced cells, no significant change was observed in any of the E1-, E2-, or E1 + E2-transduced cells for mTOR, Raptor, Rictor, and AKT. Furthermore, no significant change was detected in the phosphorylation of mTOR (S2448 or S2481) or AKT. We also verified the ratio of phosphorylated proteins for mTOR and AKT and found no significant change in the ratio of any of their phosphorylated forms over their respective pan total proteins. Results of one-way ANOVA are shown in Supplementary Table S5. These results suggested that components of the mTOR pathway may show differential sensitivity to *MECP2* gain- and loss-of-function (Figure 9).

### 3.6. Protein Translation Machinery Is Impaired in Rett Syndrome Brain Tissues but Not in MECP2E1-E2 Overexpressed Daoy Cells

**Figure 10:**
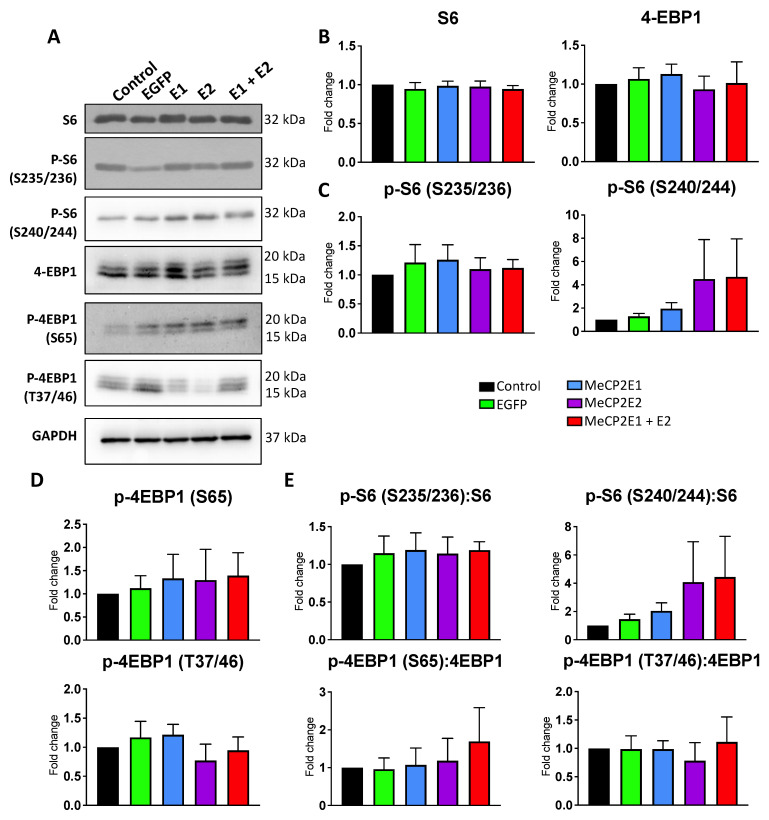
Protein analysis of ribosomal protein S6 and components of protein translation initiation in Daoy cells transduced with lentiviral vectors overexpressing MeCP2 isoforms. (**A**) Western blot analysis of the indicated proteins in non-transduced control Daoy cells, EGFP-transduced (EGFP), E1-transduced (E1), E2-transduced (E2), and E1 + E2-tranduced (E1 + E2) cells. (**B**,**C**) Quantification of the indicated total proteins from A. (**D**,**E**) Ratio analysis of phosphorylated proteins from A versus their non-phosphorylated pan proteins. *n* = 4–6 (*n* = 4 for p-4EBP1 (S65), p-S6 (235/236); *n* = 5 for p-4EBP1 (T37/46), *n* = 6 for S6, p-S6 (240/244), *n* = 6 for 4EBP1), and data are reported as mean ± SEM. For ratio of phosphorylated proteins to their pan protein abundances (Part **D**), samples from the same biological replicate were analyzed against the pan proteins from the same set. For the reported ratios, where *n* is different for the phosphorylated proteins versus their pan protein, *n* for the ratio would be equal to the lowest *n* within the phospho-pan protein pair.

It has been reported that *MECP2*-deficient human neurons display global impairment in protein translation [6]. However, the role of individual isoforms in this regard is not fully clear. In order to study whether increased MeCP2 levels would affect the components of protein translation machinery, we performed WB experiments with non-transduced Daoy cells along with the EGFP-, E1-, E2-, and E1 + E2-transduced cells. We probed the extracted protein from these cells with specific antibodies against the ribosomal protein S6, phospho-S235/236-S6 (p-S6 (S235/236)), phospho-S2240/244-S6 (p-S6 (S240/244)), 4-EBP1, phospho-S65-4EBP1 (p-4EBP1 (S65)), and phospho-T37/46-4EBP1 (p-4EBP1 (T37/46)). Compared with non-transduced or EGFP-transduced Daoy cells, no significant change was observed in E1-, E2, or E1 + E2-transduced cells for any of the tested proteins, their phosphorylated forms, or the ratio of phosphorylated proteins over their respective total pan proteins (Figure 10). Results of one-way ANOVA are shown in Supplementary Table S5. These results suggest that overexpression of MeCP2 isoforms may not have any significant impact on protein translation initiation or that Daoy cells have adapted to the increased level of MeCP2E1 and MeCP2E2 isoforms to maintain their protein translation initiation.

As MeCP2 is a chromatin-binding protein, we tested the possibility that the global chromatin structure may be altered by increased MeCP2 levels. Thus, we tested the level of specific markers of euchromatin (H3K4me3), facultative heterochromatin (H3K27me3), and constitutive heterochromatin (H3K9me3) [1,44]. No significant change was detected for E1-, E2-, or E1 + E2-transdcued cells compared with non-transduced or EGFP-transduced control cells (Supplementary Figure S5). Results of one-way ANOVA are shown in Supplementary Table S5. This suggested that the Daoy cells in culture may have adapted to the increased level of MeCP2E1 and MeCP2E2 isoforms to maintain their chromatin structure.

**Figure 11:**
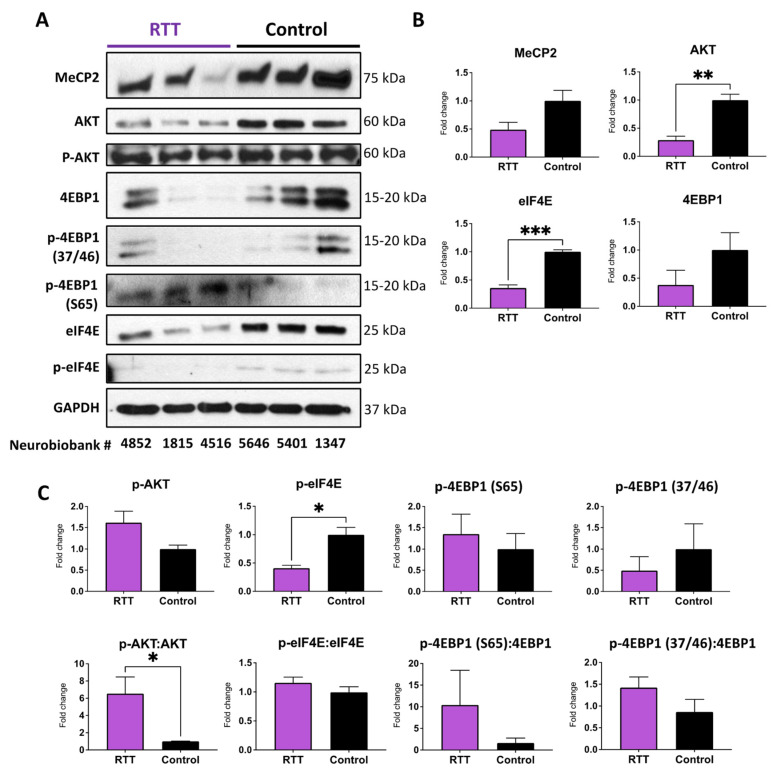
Protein analysis of indicated proteins in the brain of Rett Syndrome patients and non-Rett Syndrome controls. (**A**) Western blot analysis of the indicated proteins in the post-mortem human brain tissues (frontal cerebrum) compared with controls. The NIH NeuroBioBank ID of the samples are provided below the Western blot images. For detailed information about these samples, please refer to Table 4. (**B**) Quantification of the indicated total proteins from A. *n* = 3, and data are reported as mean ± SEM. (**C**) Quantification of the indicated phosphorylated proteins from A, as well as the ratio analysis of the phosphorylated proteins from A versus their own non-phosphorylated pan proteins, *: *p* < 0.05, **: *p* < 0.01, ***: *p* < 0.001.

Next, we tested the brain tissues of RTT patients compared with non-RTT control brain tissues. Extracted protein samples were subjected to WB with antibodies against MeCP2, AKT, p-AKT, 4EBP1 and phosphorylated forms, and eIF4E and its phosphorylated form. Our results indicated that while MeCP2 protein is reduced in RTT brains, the detected change is not statistically significant (Figure 11). This could be due to the heterogenicity of the results in RTT patients with different mutations (please see information in Table 4). Regardless, this was in agreement with our recent studies in the brain of RTT patients who showed significantly reduced *MECP2*, *MECP2E1*, and *MECP2E2* transcript levels compared with non-RTT controls, without any significant change in the MeCP2E1 and MeCP2E2 proteins detected by WB [23,39]. In contrast, reduced AKT levels in RTT brains compared with non-RTT controls were significant. When comparing the ratio of p-AKT:AKT, we noticed a significant increase in RTT patients, suggesting that the brain of RTT patients demands a higher level of phosphorylation efficiency in order to control this important signaling pathway (Figure 11). RTT patients further showed significant reduction in eIF4E. This was associated with significant reduction in p-eIF4E in RTT patients, pointing towards reduced level of protein translation initiation (Figure 11). Although the ratio of p-eIF4E:eIF4E remained similar in RTT patients compared with non-RTT controls, our results indicated a lower level of protein translation initiation in RTT brain tissues, simply due to the lower level of the available eIF4E molecules. In general, eIF4E can become phosphorylated once it is released from its binding molecule 4EBP1. This is associated with 4EBP1 phosphorylation at multiple sites including p-4EBP1 (T37/46), and the last phosphorylation site being the p-4EBP1 (S65) [45,46]. Analysis of 4EBP1 and its phosphorylated forms indicated a trend of reduced 4EBP1 and p-4EBP1 (T37/46) in the brain of RTT patients, while no obvious trend of change was observed for p-4EBP1 (S65). Although reduced 4EBP1 in RTT patients and increased phosphorylation in these tested brain tissues were statistically non-significant, they suggest an inhibition of protein translation initiation through mechanisms that may partially involve 4EBP1. Taken together, our results suggest that protein translation initiation is differently impacted by *MECP2* loss- and gain-of-function in RTT brain tissues and in the in vitro cellular model of overexpressed *MECP2* isoforms.

## 4. Discussion

MeCP2 is a multi-functional transcriptional regulator with key roles in brain development, acting in a dose-dependent manner [47]. Accordingly, its gain- or loss-of-function is associated with human disease and neurodevelopmental disorders, with compromised structural and functional properties of different brain cell types [44,47,48]. Studies have shown that inverse changes in gene expression occur in mice overexpressing or deficient in MeCP2, but also changes unique to different *MECP2* mutations have been shown [8,49,50,51]. While much progress has been made in understanding MeCP2 interactors and targets of expression, an important aspect requiring further studies remains, in understanding the functional role of the two MeCP2E1 and E2 isoforms. While MeCP2E1 is the major protein isoform expressed in the brain, there is still evidence of MeCP2E2 being expressed in the brain, with both protein variants having unique and redundant functional properties [17,52,53]. Within our study, an in vitro model overexpressing each isoform was established to test whether their individual or combined gain-of-function in a human brain cell line would provide an insight into their differential role in transcriptional gene regulation. This was subsequently complemented with studies on cell signaling pathways. Our results indicated that MeCP2E2 activates *BDNF* transcriptional expression, while both isoforms are able to significantly increase *BDNF* steady-state transcript levels. However, we observed that Daoy cells managed to prevent such induced transcriptional expression of *BDNF* to be translated into an increased level of the functional BDNF protein. This suggested additional regulatory check points are in place for regulating BDNF.

In our study, MeCP2 isoform-specific overexpression was achieved by in vitro lentiviral transduction. The Daoy medulloblastoma human cell line was selected for this study. These cells are originated from a tumor in the cerebellum of a 4-year-old boy. The cells express MeCP2 endogenously and have been used previously to study MeCP2 regulation and screen potential therapeutic targets for MeCP2-related disorders [40,41]. Lentiviral transduction of Daoy cells proved to be an efficient method for overexpressing the MeCP2 isoforms and for studying changes in nascent RNA transcripts. Expression of overexpressed MeCP2 isoforms was retained in the nucleus, which is its endogenous sub-cellular compartment.

Our studies with MG132 indicated that the MeCP2E1 protein can indeed be produced in E1 + E2-transduced cells, but it is subjected to degradation. A robust signal was detected for MeCP2E1 within an hour of MG132 treatment, suggesting activation of specific cellular mechanisms involving the proteosome pathway. However, further studies are required to fully establish the mechanisms of MeCP2E1 degradation in E1 + E2-transduced cells. Some level of increase in MeCP2E2 was also observed following MG132 treatment in E1 + E2-transduced cells, suggesting that these cells were trying to avoid an additional level of MeCP2 isoforms. Daoy cells are of cancerous origin; thus, they may adapt to MeCP2 increase and to keep their chromatin structure in a functional state. Our analysis of specific histone post-translational modifications indeed confirmed non-significant differences in specific markers of euchromatin (H3K4me3), facultative heterochromatin (H3K27me3), and constitutive heterochromatin (H3k9me3) (Supplementary Figure S3).

Rett Syndrome is a severe disorder with a wide range of neurological symptoms that engage different parts of the brain. RTT brain presents an accumulation of cellular dysfunction in neurons and glia from multiple brain regions. Currently, the full extent of these cellular and molecular damages in RTT brains is unclear. For our studies, we selected human brain cells and/or brain tissues from the cerebellum and frontal cerebrum. RTT patients show ataxia, a symptom that involves the cerebellum. In the cerebellum, *MECP2* loss-of-function mutation leads to impaired mTOR signaling [25]. Thus, Daoy cells originated from the cerebellum provide a feasible in vitro model for studying the effects of *MECP2E1-E2* gain-of-function mutations on mTOR signaling and S6 phosphorylation. Protein translation is a fundamental cellular function that is largely controlled by the mTOR-S6 signaling [26]. Thus, we tested the effects of *MECP2E1-E2* gain-of-function on the same in vitro cellular model. Whether or not *MECP2E1-E2* overexpression is associated with any change in the rate or efficiency of protein translation in these cells warrants further investigations.

Comparing our data with those in the published literature, there is evidence of overall change in protein translation in human RTT neurons, where Li et al. reported in 2013 that global protein translation is impaired in *MECP2*-deficient ESC-derived neurons [6]. These cells in nature are more like neurons of the frontal cerebrum; hence, we selected the RTT frontal cerebrum to study the components of protein translation machinery in patient brain tissues.

We further evaluated the effect of MeCP2 overexpression on cell viability by MTT assay and found that Daoy cell viability was not severely impacted by MeCP2 overexpression. Overexpression of either isoform had a slight effect on cell viability at an early time point, but their cellular viability improved over subsequent time points. In previous studies, MeCP2E1 overexpression reduced stem cell proliferation [54], and overexpression of MeCP2E2 promoted apoptosis in cerebellar rat granule cells [42]. This may represent an adverse effect present in the brain cells of patients suffering from *MECP2* duplication syndrome, but further studies would be required to establish such a hypothesis.

Our results indicated that overexpression of MeCP2E1 did not induce *MECP2E2* transcripts or vice versa in initial analyses of cells collected at non controlled time points, nor when the nascent transcript expression was analyzed at controlled time points. BDNF belongs to the family of neurotrophin factors with critical roles in neuronal differentiation, cellular growth, maintenance, and survival [55]. Indeed, BDNF deregulation has been associated with neurodevelopmental, neurological, and neurodegenerative disorders including RTT, Alzheimer’s disease, Parkinson’s disease, depression, and drug addiction. Regulation of the *BDNF* gene is complex, with 11 exons and 9 functional promoters. The human *BDNF* gene has a 3′ exon containing the coding sequence, and it is alternatively spliced with different 5′ exons to produce an array of transcripts [56]. Our study assessed changes in total *BDNF* transcripts following *MECP2E1* or *MECP2E2* overexpression, detecting that overexpression of either isoform in Daoy cells led to increased total *BDNF* transcripts. Increased MeCP2 had been linked to increased *BDNF* transcripts previously but has not been shown at the nascent level or isoform-specific levels [8]. The changes in the nascent transcripts were seen to be slightly more subtle than the increases detected in steady-state transcripts. The steady-state RNA also included the transcripts that were present in the cells prior to EU-labeling was initiated but could also represent increased *BDNF* transcript stability. Future studies of transcript stabilities in MeCP2-overexpressing cells could characterize this further. The highest fold changes in *BDNF* were seen in *MECP2E2*-transduced cells, which were at times significantly greater compared with *MECP2E1*-transduced cells. MeCP2-overexpressing cells had increased levels of nascent *BDNF* at the earlier time points compared with control cells, but this was diminished by 24 h. This could be an indication of a feedback loop reducing *BDNF* transcription.

The microRNA *miR-132* has been reported to negatively regulate MeCP2 levels in primary cortical and hippocampal rat neurons, and that decreasing MeCP2 leads to an increase in *miR-132* levels in primary hippocampal neurons [57,58]. Here, we assessed the impact of MeCP2 overexpression on *miR-132,* as it is proposed to form a homeostatic regulatory network with MeCP2 and BDNF whereby MeCP2 activates BDNF, which in turn induces *miR-132*, which then inhibits MeCP2. We did not detect any significant effect from either MeCP2E1 or MeCP2E2 on *miR-132-3p* expression levels at the nascent or steady-state levels, despite of a trend of decreased expression in the transduced cells that included EGFP-transduced cells as well (Figure 6B). This could be attributed to the fact that Daoy cells are of cerebellar origin, in agreement with a recent study from our team that we reported *miR-132-3p* transcripts are not impacted in RTT patient cerebellar brain tissues [23]. A recent study from our lab on this regulatory network in post-mortem human RTT patient brain tissues reported decreased *MECP2* and *BDNF* transcripts in the frontal cerebrum, amygdala, hippocampus, and cerebellum. The frontal cerebrum, amygdala, and hippocampus also had decreased *miR-132* transcripts, but *miR-132* transcripts were unchanged in the cerebellum. The expression of *MECP2* is highest in the cerebellum, correlating with low expression of *miR-132* in the same brain region [23]. The *miR-132-3p* strand of the microRNA was observed to be the major strand expressed in the human brain tissues, which was also observed in Daoy cells in this study. In this current study, Daoy cells overexpressing MeCP2 isoforms had increased *BDNF* transcripts, but *miR-132* was unchanged, indicating this homeostasis network may not function in human cerebellum cells in the same way suggested by studies in the murine brain. We observed changes in *miR-132,* but these were also detected in EGFP-transduced cells, indicating that it may not have been a specific effect of MeCP2 overexpression. This appears to indicate consistency with what was observed in the human RTT brain tissues.

Nucleolin and *rRNAs* are critical factors in ribosome biogenesis and cellular protein translation, which are impaired in RTT neurons. In our study, *Nucleolin* nascent and steady-state transcripts remained unchanged in Daoy cells overexpressing MeCP2 isoforms, except for a significant decrease in steady-state transcripts in E2-transduced cells at the 24 h time point. Changes in Nucleolin in previous studies have been observed primarily at the protein level, which indicates MeCP2 may impact Nucleolin post-translationally. Here, our data showed no significant differences in the Nucleolin protein or transcript in the transduced Daoy cells, irrespective of the transduced *MECP2* isoform. Ribosomal RNA transcript levels did not appear to be consistent, and they were significantly changed in some cases in MeCP2-overexpressing Daoy cells. The transcripts which showed a significant change from both non-transduced and EGFP-transduced controls included decreased *18S rRNA* nascent transcripts in E2- and E1 + E2-transduced cells at the 1 h time point. Ribosomal protein and *RNA* genes were found to be downregulated in *MECP2* knockout human embryonic stem cell-derived neurons [6]. In mice, *Mecp2* loss- and gain-of-function mutations lead to inverse gene expression changes but can also lead to gene expression changes in the same direction [10]. This may be the case for *rRNA* genes as well.

Some research evidence points towards unique function of MeCP2 isoforms. MeCP2E1 is the dominant protein isoform expressed in the brain [16,17]. MeCP2E1-specific loss-of-function is sufficient to cause RTT-like symptoms, while E2-specific mutations have not been observed in patients so far [14,59,60]. Mice lacking the MeCP2E2 isoform display no adverse neurological symptoms; however, a reduction in embryonic viability has been observed [61]. Mice with the MeCP2E1-specific mutation develop RTT phenotypes [62]. Expression of the MeCP2E2 alone is capable of ameliorating RTT symptoms in *Mecp2*-null mice [63,64]. Mice with the E1-specific mutation showed an increased level of E2, although not enough to rescue the mice [62]. Differential expression of these isoforms during neuronal differentiation has been shown in vitro in murine differentiated neural stem cells [21] and in vivo, where MeCP2E1 and MeCP2E2 increase during mouse brain development, with MeCP2E1 showing an earlier onset of expression [17]. MeCP2E1 protein expression was found to be uniform across different mouse brain regions, while MeCP2E2 protein showed a differential expression pattern in different parts of the brain [17]. Studies which have assessed gene expression changes in *Mecp2*-mutant mice have used mice with knockdown or overexpression of both isoforms [8,9,10].

It has been reported that *MECP2* loss-of-function mutation leads to impaired mTOR signaling, with significant overactivation of mTOR signaling (phosphorylation of mTORC1 and/or mTORC2) compared with controls [25]. Our assessment of transduced Daoy cells suggested marginal but non-significant impact on the mTOR pathway, suggesting that the brain cells may have differential sensitivity to *MECP2* gain- and loss-of-function. In vitro cultured RTT neurons show impaired protein translation [6], and our data confirm that in human RTT brain tissues, components of the protein translation machinery are compromised. This is associated with a significant reduction in eIF4E protein in RTT patients. Additionally, eIF4E release from its binding to the 4EBP1–eIF4E complex may have been affected. Therefore, our data points towards reduced protein translation initiation through reduced phosphorylation of the eIF4E that would significantly affect the subsequent steps of protein translation. Interestingly, transduced Daoy cells with increased level of MeCP2 isoforms did not show signs of compromised protein translation initiation, suggesting that the protein translation initiation may be differently impacted by *MECP2* loss- and gain-of-function. The human RTT brain tissues were from patients who carried common (NIH NeuroBioBank #4516: R255X) and uncommon (NIH NeuroBioBank #1815: IVS3-2A > G; NIH NeuroBioBank# 4852: G451T) *MECP2* mutations. Therefore, one should be cautious in extending our results to all RTT patients, as the brain tissues may be differently affected by *MECP2* mutations in various functional domains of the MeCP2 protein. One limitation of our study is a side-by-side comparison of the post-mortem brain tissues from RTT and MDS patients. However, access to the human brain tissues is limited, and MDS frequency is much rarer than RTT, which also is a rare disease itself. Future directions would include studies of the nascent protein synthesis in RTT and MDS model systems and how MeCP2E1 and MeCP2E2 homeostasis regulation is involved in mTOR signaling, ribosome biogenesis, and protein translation initiation.

## 5. Conclusions

In conclusion, Daoy cells overexpressing MeCP2 isoforms were successfully established, validated, and characterized. A significant increase in *BDNF* transcription/transcript levels was observed in E1- and E2-overexpressing cells, without any significant change in *miR-132*, suggesting that the MeCP2-BDNF-*miR-132* regulatory network may not be present in these cells. Ribosomal RNA transcript results were more dynamic, with significant decreases in *18S rRNA* nascent transcripts in E2- and E1 + E2-transduced cells at specific time points. MG132 studies suggested that MeCP2E1 is susceptible to proteosome degradation when an increased level of E2 is present. We observed that Daoy brain cells could adapt to the overexpression of E1 and E2 at different levels. These included the status of chromatin structure, mTOR signaling, AKT phosphorylation, and components of protein translation initiation. This was in contrast with the results that we obtained with the human RTT brain tissues with *MECP2* loss-of-function mutation(s). Our data suggest that different sensitivity of the human brain cells may exist regarding protein translation initiation machinery and mTOR signaling to *MECP2* gain- and loss-of-function. Taken together, our results provide important insight into the dose-dependent MeCP2 function, as well as the RTT and MDS mechanism of disease.

## Figures and Tables

**Table 1:** Primary antibodies used in Western blot and immunofluorescence.

Primary Antibody	Application and Dilution	Description	Source
c-Myc	WB 1:1000	mouse monoclonal	Thermo Fisher Scientific, A21280
c-Myc	IF 1:50	mouse monoclonal	Thermo Fisher Scientific, MA5-12080
MeCP2E1	WB, IF 2 μg/mL	chicken polyclonal	Custom-made (Rastegar lab) [16,17]
MeCP2E2	WB, IF 2 μg/mL	chicken polyclonal	Custom-made (Rastegar lab) [17]
MeCP2 (C-terminal)	WB 1:1000, IF 1:200	rabbit polyclonal	Thermo Fisher Scientific, PA5-12234
GAPDH	WB 1:7500	mouse monoclonal	Santa Cruz Biotechnology, SC-47724
eIF4E	WB 1:2000	rabbit monoclonal	Sigma, E5906
p-eIF4E	WB 1:1000	rabbit monoclonal	Sigma, SAB4504389
4E-BP1	WB 1:1000	rabbit monoclonal	Cell Signaling Technology, 9644
p-4EBP1	WB 1:1000	rabbit monoclonal	Cell Signaling Technology, 2855 and 9451
MeCP2	WB 1:1000	rabbit monoclonal	Cell Signaling Technology, 3456
AKT	WB 1:1000	rabbit monoclonal	Cell Signaling Technology, 9272S
p-AKT	WB 1:500	rabbit monoclonal	Cell Signaling Technology, 2965S

**Table 2:** List of secondary antibodies used in Western blot and immunofluorescence.

Secondary Antibody	Application and Dilution	Source
HRP-conjugated anti-rabbit IgG	WB 1:5000	Sigma A6154
Peroxidase-AffiniPure sheep anti-mouse IgG	WB 1:7500	Jackson ImmunoResearch 115-035-174
Peroxidase-AffiniPure goat anti-chicken IgY	WB 1:7500	Jackson ImmunoResearch 103-035-155
Alexa Fluor 594 conjugated goat anti-chicken IgY	IF 1:1000	Invitrogen, Thermo Fisher Scientific, A11042
Alexa Fluor 594 conjugated goat anti-rabbit IgG	IF 1:1000	Invitrogen, Thermo Fisher Scientific, A11037
Alexa Fluor 488 conjugated goat anti-mouse IgG	IF 1:1000	Invitrogen, Thermo Fisher Scientific, A11017
Anti-Rabbit IgG Peroxidase antibody produced in goat	WB 1:5000	Sigma, A6154
Peroxidase AffiniPure Goat Anti-Mouse IgG	WB 1:7000	Jackson ImmunoResearch Laboratories, 115-035-174

**Table 3:** Primer Sequences Used for qRT-PCR.

Primer Name		Sequence	References
*GAPDH*	Forward	5′-CCACTCCTCCACCTTTGAC-3′	[23]
	Reverse	5′-ACCCTGTTGCTGTAGCCA-3′	
*MECP2E1*	Forward	5′-AGGAGAGACTGGAAGAAAAGTC-3′	[23]
	Reverse	5′-CTTGAGGGGTTTGTCCTTGA-3′	
*MECP2E2*	Forward	5′-CTCACCAGTTCCTGCTTTGATGT-3′	[23]
	Reverse	5′-CTTGAGGGGTTTGTCCTTGA-3′	
*BDNF*	Forward	5′-TAACGGCGGCAGACAAAAAGA-3′	[23]
	Reverse	5′-GAAGTATTGCTTCAGTTGGCCT-3′	
*Nucleolin*	Forward	5′-AGCAAAGAAGGTGGTCGTTT-3′	[25]
	Reverse	5′-CTTGCCAGGTGTGGTAACTG-3′	
*45S rRNA*	Forward	5′-CTCCGTTATGGTAGCGCTGC-3′	[25]
	Reverse	5′-GCGGAACCCTCGCTTCTC-3′	
*28S rRNA*	Forward	5′-AGAGGTAAACGGGTGGGGTC-3′	[25]
	Reverse	5′-GGGGTCGGGAGGAACGG-3′	
*18S rRNA*	Forward	5′-GATGGTAGTCGCCGTGCC-3′	[25]
	Reverse	5′-GCCTGCTGCCTTCCTTGG-3′	

**Table 4:** Characteristics of Rett Syndrome patients and controls.

NIH NeuroBioBank Sample Number	*MECP2* Mutation	Sex	Age (Years)	References
4852	G451T	Female	19	[25]
1815	IVS3-2A>G	Female	18	
4516	R255X	Female	20	[23,25,39]
5646	Non-RTT Control	Female	20	[25,39]
5401	Non-RTT Control	Female	18	[23,39]
1347	Non-RTT Control	Female	19	[25]

## Data Availability

All information related to the generated data exists within the manuscript.

## Supplementary Materials

The following supporting information can be downloaded at: https://www.mdpi.com/article/10.3390/cells11091442/s1, Table S1: Two-way ANOVA results applicable to Figure S2 (MTT assay); Table S2: Two-way ANOVA results for Nascent RNA (applicable to Figure 4, Figure 6, Figure 7 and Figure 8); Table S3: Two-way ANOVA results for Total RNA (applicable to Figure 4, Figure 6, Figure 7 and Figure 8); Table S4: Two-way ANOVA results applicable to Figure 5 (MTT assay for MG132); Table S5: One-way ANOVA results applicable to protein levels in Figure 6, Figure 7, Figure 9 and Figure 10; Figure S1: Immunofluorescence imaging of MeCP2 and c-Myc in transduced Daoy cells; Figure S2: MTT cell viability assay of Daoy cells transduced with lentiviral vectors expressing EGFP or overexpressing *MECP2E1* and *MECP2E2* isoforms; Figure S3: Protein analysis of indicated histone modifications in Daoy cells transduced with lentiviral vectors expressing MeCP2 isoforms.
