# Supplementary material for: Differential Sensitivity of the Protein Translation Initiation Machinery and mTOR Signaling to MECP2 Gain- and Loss-of-Function Involves MeCP2 Isoform-Specific Homeostasis in the Brain
Source: Cells. 2022 Apr 24;11(9):1442. doi: 10.3390/cells11091442 (PMC9105805; doi:10.3390/cells11091442)
Supplement: Supplementary file 1 [file cells-11-01442-s001.zip › cells-1649278-supplementary.pdf]

Supplementary Table S1. Two Way ANOVA results applicable to Figure S2 (MTT assay).

ANOVA table	SS	DF	MS	F (DFn, DFd)	P value	P value summary
Interaction	2839	8	354.9	F (8, 75) = 3.503	P=0.0017	**
Transduction	2045	4	511.3	F (4, 75) = 5.048	P=0.0012	**
Time	6005	2	3003	F (2, 75) = 29.64	P<0.0001	****
Residual	7597	75	101.3			

Supplementary Table S2. Two Way ANOVA results for Nascent RNA (applicable to Figures 4, 6, 7, 8).

Gene Target	*ANOVA table*	SS	DF	MS	F (DFn, DFd)	P value	P value summary
MECP2E1	Interaction	1275	8	159.4	F (8, 30) = 1.428	P=0.2255	ns
	Time	358.5	2	179.2	F (2, 30) = 1.606	P=0.2175	ns
	Transduction	9167	4	2292	F (4, 30) = 20.53	P<0.0001	****
	Residual	3348	30	111.6			
MECP2E2	Interaction	879.6	8	109.9	F (8, 30) = 10.05	P<0.0001	****
	Time	543.7	2	271.9	F (2, 30) = 24.85	P<0.0001	****
	Transduction	8727	4	2182	F (4, 30) = 199.4	P<0.0001	****
	Residual	328.2	30				
BDNF	Interaction	1.351	8	0.1689	F (8, 30) = 1.056	P=0.4187	ns
	Time	1.673	2	0.8363	F (2, 30) = 5.228	P=0.0113	*
	Transduction	10.87	4	2.718	F (4, 30) = 16.99	P<0.0001	****
	Residual	4.800	30	0.1600			
miR132	Interaction	0.3935	8	0.04919	F (8, 30) = 0.3417	P=0.9423	ns
	Time	0.2091	2	0.1045	F (2, 30) = 0.7263	P=0.4920	ns
	Transduction	2.525	4	0.6313	F (4, 30) = 4.386	P=0.0065	**
	Residual	4.318	30	0.1439			
Nucleolin	Interaction	0.04465	8	0.005581	F (8, 30) = 0.4468	P=0.8829	ns
	Time	0.04355	2	0.02178	F (2, 30) = 1.743	P=0.1922	ns
	Transduction	0.06698	4	0.01675	F (4, 30) = 1.340	P=0.2779	ns
	Residual	0.3748	30	0.01249			
45S rRNA	Interaction	3.241	8	0.4052	F (8, 30) = 0.5608	P=0.8010	ns
	Time	2.321	2	1.161	F (2, 30) = 1.606	P=0.2174	ns
	Transduction	1.308	4	0.3269	F (4, 30) = 0.4525	P=0.7697	ns
	Residual	21.67	30	0.7225			
28S	Interaction	0.4836	8	0.06046	F (8, 30) = 0.6086	P=0.7633	ns
	Time	0.1530	2	0.07648	F (2, 30) = 0.7699	P=0.4720	ns
	Transduction	0.5779	4	0.1445	F (4, 30) = 1.454	P=0.2407	ns

	Residual	2.980	30	0.09934			
18S	Interaction	0.1329	8	0.01661	F (8, 30) = 1.274	P=0.2935	ns
	Time	0.02695	2	0.01347	F (2, 30) = 1.034	P=0.3680	ns
	Transduction	0.4643	4	0.1161	F (4, 30) = 8.905	P<0.0001	****
	Residual	0.3910	30	0.01303			

Supplementary Table S3. Two Way ANOVA results for Total RNA (applicable to Figures 4, 6, 7, 8).

Gene Target	*ANOVA table*	SS	DF	MS	F (DFn, DFd)	P value	P value summary
MECP2E1	Interaction	39.05	8	4.882	F (8, 30) = 0.3648	P=0.9309	ns
	Time	17.08	2	8.538	F (2, 30) = 0.6380	P=0.5354	ns
	Transduction	5497	4	1374	F (4, 30) = 102.7	P<0.0001	****
	Residual	401.5	30	13.38			
MECP2E2	Interaction	24.13	8	3.016	F (8, 30) = 0.2649	P=0.9725	ns
	Time	8.667	2	4.333	F (2, 30) = 0.3807	P=0.6867	ns
	Transduction	8869	4	2217	F (4, 30) = 194.8	P<0.0001	****
	Residual	341.5	30	11.38			
BDNF	Interaction	2.298	8	0.2872	F (8, 30) = 6.178	P=0.0001	***
	Time	3.304	2	1.652	F (2, 30) = 35.53	P<0.0001	****
	Transduction	19.86	4	4.965	F (4, 30) = 106.8	P<0.0001	****
	Residual	1.395	30	0.04649			
miR132	Interaction	1.175	8	0.1469	F (8, 30) = 0.4395	P=0.8876	ns
	Time	1.700	2	0.8498	F (2, 30) = 2.543	P=0.0955	ns
	Transduction	1.356	4	0.3390	F (4, 30) = 1.014	P=0.4157	ns
	Residual	10.03	30	0.3342			
Nucleolin	Interaction	0.05647	8	0.007058	F (8, 30) = 0.7062	P=0.6838	ns
	Time	0.004821	2	0.002411	F (2, 30) = 0.2412	P=0.7872	ns
	Transduction	0.2394	4	0.05986	F (4, 30) = 5.988	P=0.0012	**
	Residual	0.2999	30	0.009995			
45S rRNA	Interaction	3.231	8	0.4039	F (8, 30) = 0.6056	P=0.7657	ns
	Time	9.146	2	4.573	F (2, 30) = 6.858	P=0.0035	**
	Transduction	2.860	4	0.7150	F (4, 30) = 1.072	P=0.3876	ns
	Residual	20.00	30	0.6668			
28S rRNA	Interaction	2.089	8	0.2611	F (8, 30) = 0.6435	P=0.7352	ns
	Time	5.574	2	2.787	F (2, 30) = 6.868	P=0.0035	**
	Transduction	2.012	4	0.5031	F (4, 30) = 1.240	P=0.3152	ns
	Residual	12.17	30	0.4058			

18S rRNA	Interaction	0.3190	8	0.03988	F (8, 30) = 0.6168	P=0.7568	ns
	Time	0.1949	2	0.09746	F (2, 30) = 1.507	P=0.2378	ns
	Transduction	1.032	4	0.2581	F (4, 30) = 3.992	P=0.0103	*
	Residual	1.940	30	0.06465			

Supplementary Table S4. Two Way ANOVA results applicable to Figure 5 (MTT assay for MG132).

ANOVA table	**SS**	**DF**	**MS**	**F (DFn, DFd)**	**P value**	**P value summary**
Interaction	6889	8	861.1	F (8, 60) = 33.03	P<0.0001	****
Time	23914	2	11957	F (2, 60) = 458.7	P<0.0001	****
MG132 Concentration	12605	4	3151	F (4, 60) = 120.9	P<0.0001	****
Residual	1564	60	26.07			

Supplementary Table S5. One Way ANOVA results applicable to protein levels in Figures 6, 7, 9, and 10.

Protein	***ANOVA table***	**SS**	**DF**	**MS**	**F (DFn, DFd)**	**P value**	**P value summary**
Figure 6C							
BDNF (25 kDa)	Transduction	0.5978	4	0.1495	F (4, 15) = 1.480	P=0.2575	ns
	Residual	1.514	15	0.1010			
	Total	2.112	19				
BDNF (25 &30 kDa)	Transduction	0.5048	4	0.1262	F (4, 15) = 3.881	P=0.0234	*
	Residual	0.4878	15	0.03252			
	Total	0.9926	19				
BDNF (30 kDa)	Transduction	25.78	4	6.446	F (4, 15) = 1.060	P=0.4099	ns
	Residual	91.18	15	6.078			
	Total	117.0	19				
Figure 7B							
Nucleolin	Transduction	0.3613	4	0.09032	F (4, 25) = 0.2905	P=0.8813	ns
	Residual	7.771	25	0.3109			
	Total	8.133	29				
Figure 9							
mTOR	Transduction	0.4365	4	0.1091	F (4, 15) = 0.3962	P=0.8082	ns
	Residual	4.131	15	0.2754			
	Total	4.568	19				
Raptor	Transduction	0.7777	4	0.1944	F (4, 25) = 0.5746	P=0.6836	ns
	Residual	8.459	25	0.3384			
	Total	9.237	29				
Rictor	Transduction	0.2412	4	0.06030	F (4, 15) = 0.6584	P=0.6302	ns
	Residual	1.374	15	0.09158			
	Total	1.615	19				
AKT	Transduction	0.3178	4	0.07944	F (4, 25) = 0.4893	P=0.7435	ns
	Residual	4.059	25	0.1623			

	Total	4.376	29				
p-MTOR (S2481)	Transduction	1.871	4	0.4678	F (4, 15) = 0.4481	P=0.7722	ns
	Residual	15.66	15	1.044			
	Total	17.53	19				
p-mTOR (S2448)	Transduction	5.378	4	1.345	F (4, 15) = 0.4771	P=0.7521	ns
	Residual	42.27	15	2.818			
	Total	47.65	19				
p-AKT	Transduction	5.425	4	1.356	F (4, 20) = 0.8439	P=0.5138	ns
	Residual	32.14	20	1.607			
	Total	37.57	24				
p-MTOR (S2481): mTOR	Transduction	2.803	4	0.7009	F (4, 15) = 0.8255	P=0.5291	ns
	Residual	12.73	15	0.8490			
	Total	15.54	19				
p-mTOR (S2448): mTOR	Transduction	9.871	4	2.468	F (4, 15) = 0.6227	P=0.6534	ns
	Residual	59.44	15	3.963			
	Total	69.31	19				
p-AKT: AKT	Transduction	19.66	4	4.914	F (4, 20) = 0.8077	P=0.5348	ns
	Residual	121.7	20	6.084			
	Total	141.3	24				
Figure 10							
S6	Transduction	0.01487	4	0.003717	F (4, 25) = 0.1718	P=0.9508	ns
	Residual	0.5408	25	0.02163			
	Total	0.5557	29				
4-EBP1	Transduction	0.1306	4	0.03264	F (4, 25) = 0.1917	P=0.9404	ns
	Residual	4.256	25	0.1702			
	Total	4.386	29				
p-S6 (S235/236)	Transduction	0.1646	4	0.04115	F (4, 15) = 0.2303	P=0.9171	ns
	Residual	2.681	15	0.1787			
	Total	2.845	19				
p-S6 (S240/244)	Transduction	75.45	4	18.86	F (4, 25) = 0.7002	P=0.5991	ns
	Residual	673.4	25	26.94			
	Total	748.9	29				
p-4EBP1 (S65)	Transduction	0.4247	4	0.1062	F (4, 15) = 0.1285	P=0.9697	ns
	Residual	12.39	15	0.8263			
	Total	12.82	19				
p-S6 (S235/236): S6	Transduction	0.09786	4	0.02446	"F (4, 15) = 0.1849"	P=0.9426	ns
	Residual	1.984	15	0.1323			
	Total	2.082	1				
p-S6 (S240/244): S6	Transduction	58.37	4	14.59	F (4, 25) = 0.7172	P=0.5881	ns
	Residual	508.6	25	20.34			
	Total	567.0	29				
p-4EBP1 (T37/46)	Transduction	0.6387	4	0.1597	F (4, 20) = 0.6610	P=0.6263	ns
	Residual	4.831	20	0.2416			
	Total	5.470	24				
	Transduction	1.417	4	0.3542	F (4, 15) = 0.3058	P=0.8696	ns

p-4EBP1 (S65): 4EBP1	Residual	17.37	15	1.158			
	Total	18.79	19				
p-4EBP1 (T37/46): 4EBP1	Transduction	0.2865	4	0.07162	F (4, 20) = 0.1917	P=0.9399	ns
	Residual	7.471	20	0.3736			
	Total	7.758	24				
Figure S3							
H3K4Me3	Transduction	0.3499	4	0.08747	F (4, 15) = 2.340	P=0.1022	ns
	Residual	0.5606	15	0.03737			
	Total	0.9105	19				
H3K9Me3	Transduction	1.919	4	0.4796	F (4, 15) = 0.7669	P=0.5631	ns
	Residual	9.382	15	0.6255			
	Total	11.30	19				
H3K27Me3	Transduction	0.3115	4	0.07788	F (4, 15) = 0.4731	P=0.7548	ns
	Residual	2.469	15	0.1646			
	Total	2.781	19				

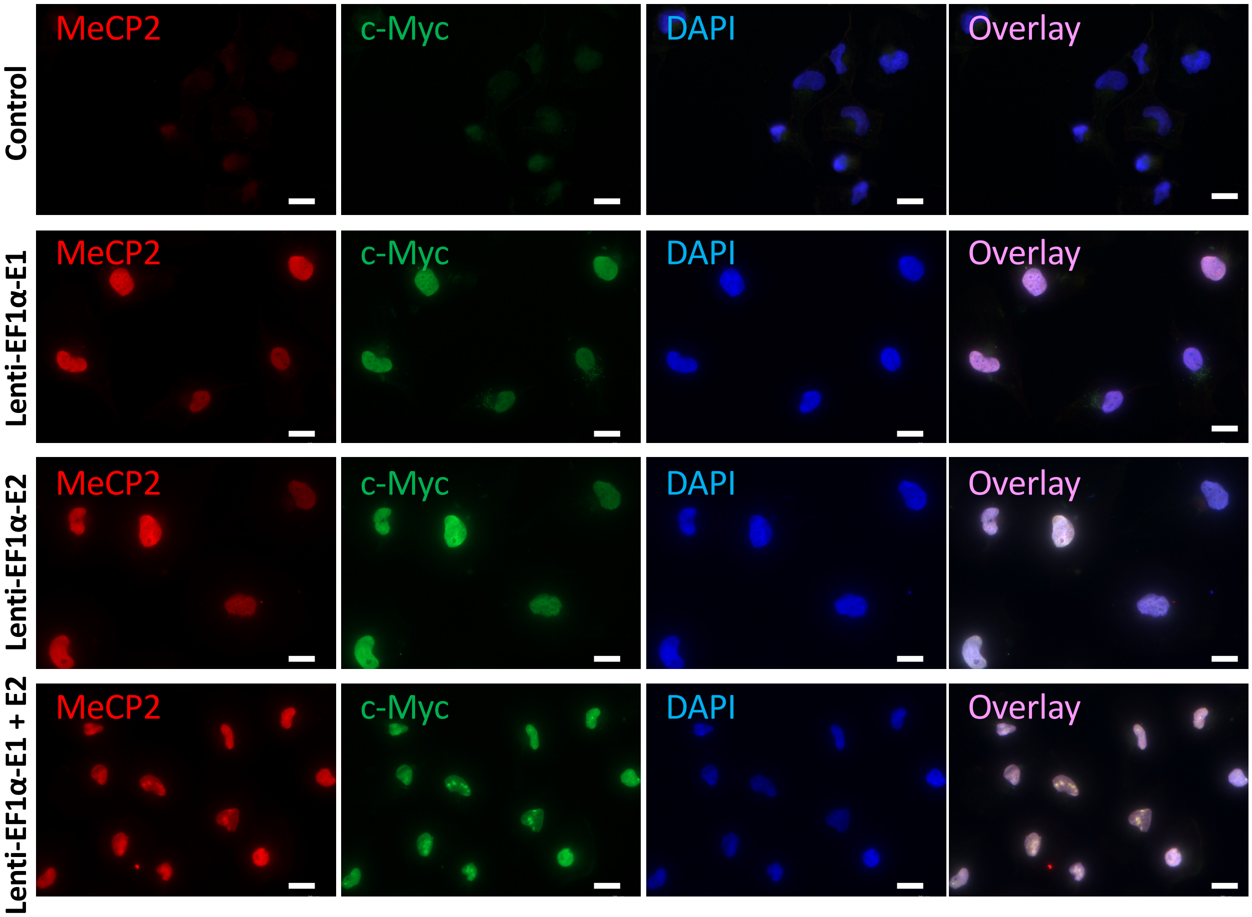

Supplementary Figure S1. Immunofluorescence imaging of MeCP2 and c-Myc in transduced Daoy cells. Images show MeCP2 (red) and c-Myc tag (green) in the nuclei of non-transduced control Daoy cells, and cells transduced with Lenti-EF1α-E1, Lenti-EF1α-E2, and Lenti-EF1α-E1+Lenti-EF1α-E2 vectors. Scale bars represents 20 μm.

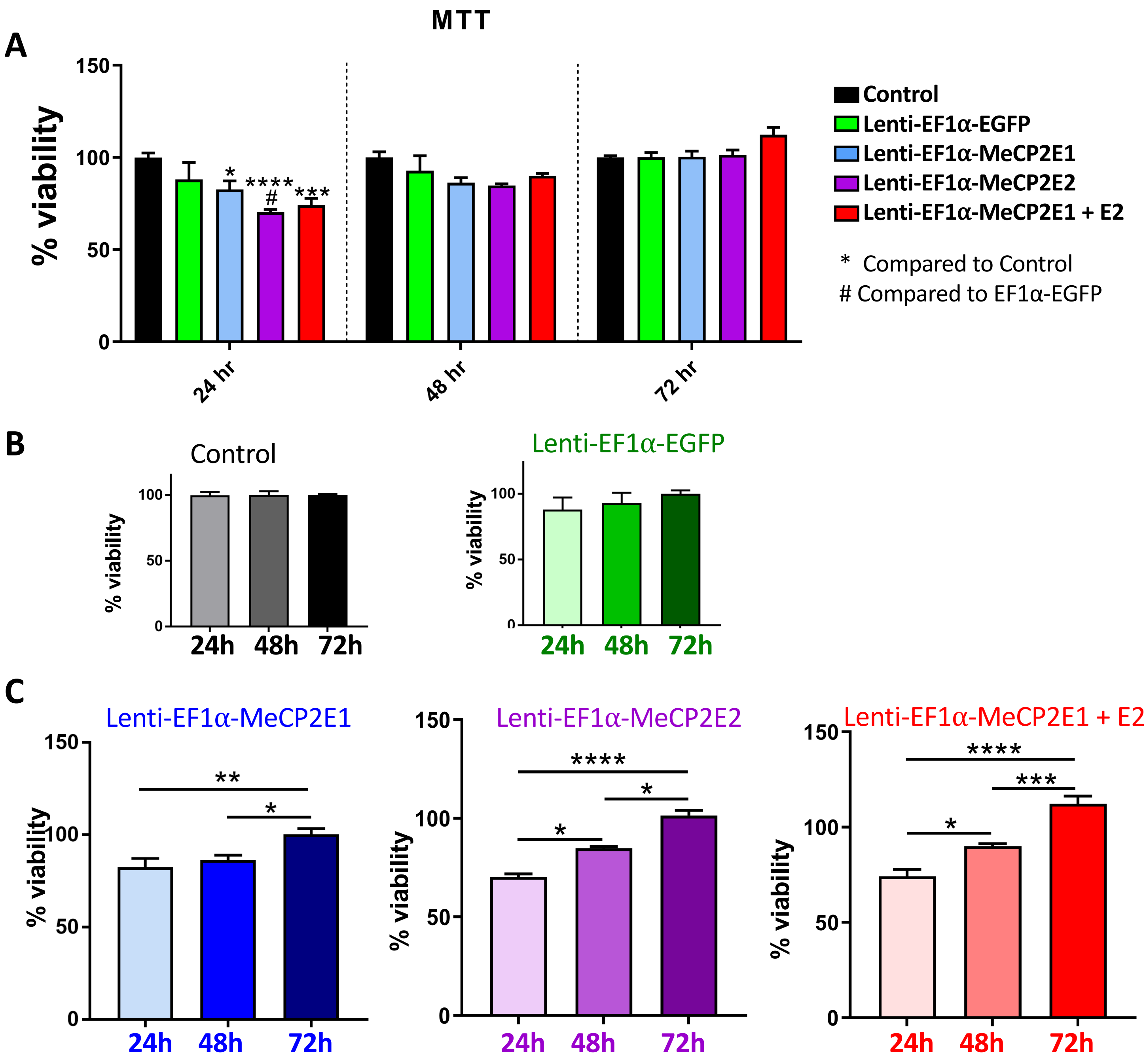

Supplementary Figure S2. MTT cell viability assay of Daoy cells transduced with lentiviral vectors expressing EGFP or overexpressing *MECP2E1* and *MECP2E2* isoforms. MTT assay was performed at 24h, 48h, and 72h following seeding and percent viability determined relative to non-transduced control Daoy cells. A) Differences in percent viability between the transduced cell types at each time point. B-C) Differences in percent viability between time points for each cell type. Experiments are done twice independently, each time in triplicates: N=2, n=6, and data are reported as mean ±SEM. Results are analyzed by two-way ANOVA followed by Tukey's multiple comparisons test. Significances from Tukey's multiple comparisons test are presented as *p<0.05, **p<0.01, ***p<0.001, and ****p<0.0001.

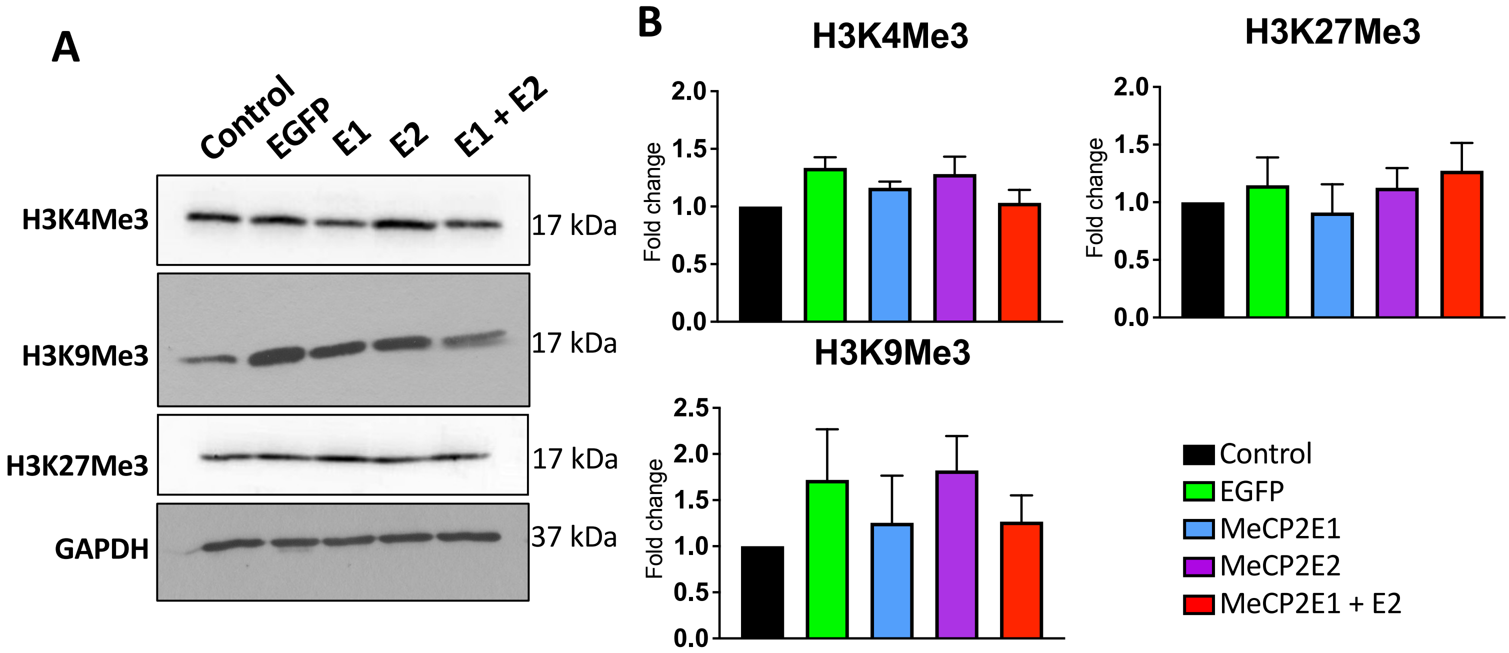

Supplementary Figure S3. Protein analysis of indicated histone modifications in Daoy cells transduced with lentiviral vectors expressing MeCP2 isoforms. A) Western blot analysis of the indicated proteins in non-transduced control Daoy cells, EGFP-transduced (EGFP), E1-transduced (E1), E2-transduced (E2) and E1+E2-tranduced (E1+E2) cells. B) Quantification of the indicated total proteins from A. N=4, and data are reported as mean ±SEM.
